# Antipsychotic use during pregnancy and risk of specific neurodevelopmental disorders and learning difficulties in children: a multinational cohort study

**DOI:** 10.1016/j.eclinm.2024.102531

**Published:** 2024-03-17

**Authors:** Claudia Bruno, Carolyn E. Cesta, Vidar Hjellvik, Sinna Pilgaard Ulrichsen, Marte-Helene Bjørk, Buket Öztürk Esen, Malcolm B. Gillies, Mika Gissler, Alys Havard, Øystein Karlstad, Maarit K. Leinonen, Mette Nørgaard, Sallie-Anne Pearson, Johan Reutfors, Kari Furu, Jacqueline M. Cohen, Helga Zoega

**Affiliations:** a School of Population Health, Faculty of Medicine and Health, University of New South Wales, Sydney, Australia; b Department of Chronic Diseases, Norwegian Institute of Public Health, Oslo, Norway; c Centre for Pharmacoepidemiology, Department of Medicine Solna, Karolinska Institutet, Stockholm, Sweden; d Department of Clinical Epidemiology, Department of Clinical Medicine, Aarhus University and Aarhus University Hospital, Aarhus, Denmark; e Department of Clinical Medicine, University of Bergen, Bergen, Norway; f Department of Neurology, Haukeland University Hospital, Bergen, Norway; g Knowledge Brokers, Finnish Institute for Health and Welfare, Helsinki, Finland; h Department of Molecular Medicine and Surgery, Karolinska Institutet, Stockholm, Sweden; i Research Centre for Child Psychiatry, University of Turku, Turku, Finland; j National Drug and Alcohol Research Centre, Faculty of Medicine and Health, University of New South Wales, Sydney, Australia; k Centre for Fertility and Health, Norwegian Institute of Public Health, Oslo, Norway; l Centre of Public Health Sciences, Faculty of Medicine, University of Iceland, Reykjavik, Iceland

**Keywords:** Antipsychotics, Pregnancy, Children, Neurodevelopmental disorders, Academic performance, Nordic health registers

## Abstract

**Background:**

Antipsychotics are commonly prescribed to treat a range of psychiatric conditions in women of reproductive age and during pregnancy, including schizophrenia, bipolar disorder, anxiety, depression, autism spectrum disorder, and insomnia. This study aimed to evaluate whether children exposed to antipsychotic medication prenatally are at increased risk of specific neurodevelopmental disorders and learning difficulties.

**Methods:**

Our population-based cohort study used nationwide register data (1 January 2000–31 December 2020) on pregnant women diagnosed with a psychiatric disorder and their live-born singletons from Denmark, Finland, Iceland, Norway, and Sweden. Cox proportional hazard regression yielded propensity score-weighted hazard ratios (aHRs) and 95% confidence intervals (CIs) for risk of intellectual-, speech or language-, learning-developmental disorders, and a composite outcome of the listed disorders. We defined poor performance as scoring within the lowest quartile on national school tests in mathematics and language arts. We estimated propensity score-weighted risk ratios (aRRs) using Poisson regression. We analysed data from Denmark separately and pooled results using random effects meta-analysis.

**Findings:**

Among 213,302 children (median follow-up: 6.7 years), 11 626 (5.5%) were exposed to antipsychotics prenatally. Adjusted risk estimates did not suggest an increased risk of neurodevelopmental disorders: aHR of 1.06 (95% CI 0.94–1.20) for the composite outcome, or for poor academic performance: aRR of 1.04 (95% CI 0.91–1.18) in mathematics, and of 1.00 (95% CI 0.87–1.15) in language arts. Results were generally consistent across individual medications, trimesters of exposure, sibling- and sensitivity analyses.

**Interpretation:**

The findings of this large multinational cohort study suggest there is little to no increased risk of child neurodevelopmental disorders or learning difficulties after prenatal exposure to antipsychotics. Our findings can assist clinicians and women managing mental illness during pregnancy.

**Funding:**

This study was funded by the 10.13039/501100004785NordForsk Nordic Program on Health and Welfare (Nordic Pregnancy Drug Safety Studies, project No. 83539), by the 10.13039/501100005416Research Council of Norway (International Pregnancy Drug Safety Studies, project No. 273366) and by the 10.13039/501100005416Research Council of Norway through its Centres of Excellence funding scheme (project No. 262700), and UNSW Scientia Programme Awards (PS46019, PS46019-A).

**Research in context**

**Evidence before this study**

Antipsychotics are increasingly used among women of reproductive age and during pregnancy. We searched PubMed database with the search terms: ((Antipsychotic) AND (prenatal)) AND (neurodevelopmental) for articles in any language published between inception and 1 August 2023. Two reviews, summarising studies published until July 2017, found that while evidence from preclinical studies consistently reported neurotoxicity of prenatal antipsychotic exposure, results from clinical studies in humans were inconsistent and unreliable. With some evidence of cognitive-, motor-, social-, emotional and adaptive impairment and delays in reaching developmental milestones, the reviews concluded that due to a lack of high-quality studies and limited sample sizes, the risk of neurodevelopmental disorders in children exposed to antipsychotics prenatally remained unclear.

More recently, three population-based cohort studies published in 2021 and 2022 suggested no increased risk for neurodevelopmental outcomes such as attention-deficit/hyperactivity disorder or autism spectrum disorder - any observed associations were considered to be due to the underlying maternal psychiatric condition. Only one of three studies, had a sample size large enough to evaluate risks associated with individual antipsychotics, finding a potential signal with aripiprazole which requires replication. Another recent population-based study from Denmark (2022), also did not find antipsychotic medication use during pregnancy to be associated with children's standardized test scores on national academic assessments, a useful marker of cognitive functioning.

**Added value of this study**

We expanded considerably on the existing evidence on long-term safety of antipsychotics by combining data from multiple countries with similar health and education systems. The large source population allowed us to examine individual antipsychotic medications and better control for confounding by maternal indication by restricting our study population to children of women with a diagnosed psychiatric disorder.

This multinational cohort study, including 213,302 children born to women with a diagnosed psychiatric disorder found no increased risk of intellectual developmental disorders, developmental speech or language disorders, developmental learning disorders, or poor academic performance among 11,626 (5.5%) children exposed to antipsychotics prenatally.

**Implications of all the available evidence**

Our study provides robust real-world evidence for the long-term safety of antipsychotic use in pregnancy, reassuringly suggesting there is little to no increased risk of child intellectual developmental disorders, developmental speech or language disorders or learning difficulties after prenatal exposure to antipsychotics.

Population-based cohort studies using large administrative or register data are essential to examine the perinatal risk factors for neurodevelopmental outcomes due to their large sample size and length of follow-up. However, as statistical noise is inevitable for very rare exposure-outcome combinations careful consideration of the evidence and corroboration between data sources is important.

## Introduction

Primarily indicated for schizophrenia and bipolar disorder, antipsychotics are commonly prescribed to treat a range of psychiatric conditions in women of reproductive age and during pregnancy, including anxiety, depression, autism spectrum disorder, and insomnia.^1^, ^2^, ^3^, ^4^, ^5^, ^6^ Similar to the trend among the general population, antipsychotic use during pregnancy has increased in recent years.^7^^,^^8^ Animal studies have demonstrated that prenatal exposure to individual antipsychotics, including haloperidol, risperidone, quetiapine, and olanzapine, can lead to neurotoxicity which could in turn lead to lasting impairments in learning and memory acquisition and retention.^9^^,^^10^ The degree to which antipsychotics cross the placenta, and accumulate in fetal lung and brain tissue varies depending on the specific medication, highlighting the importance of examining effects of individual antipsychotics.^11^

Human data on long-term neurodevelopment after prenatal exposure to antipsychotics are still sparse.^12^ Three separate population-based cohort studies accounting for maternal psychiatric conditions from the United States,^13^ Nordic countries,^14^ and Hong Kong^15^ suggested no increased risk for neurodevelopmental outcomes such as attention-deficit/hyperactivity disorder (ADHD) or autism spectrum disorder. In Denmark no association was observed between prenatal exposure to the most commonly used antipsychotics and children's scores on standardized academic tests.^16^ Nevertheless, evidence is still limited for specific neurodevelopmental disorders, and specific antipsychotic medications.

In this study, we aimed to close the evidence gap regarding the long-term safety of commonly used antipsychotics in pregnancy. We conducted the most comprehensive study to date by combining nationwide data from five Nordic countries and robustly accounted for underlying maternal psychiatric disorder and other confounding factors. We assessed risks of child neurodevelopmental disorders and learning difficulties—measured as diagnoses of intellectual, learning and speech or language disorders, poor academic performance in mathematics and language arts after prenatal exposure to any antipsychotic and individual antipsychotics.

## Methods

### Study design, data sources and study cohort

We conducted a population-based cohort study including all live-born singletons in Denmark (1 January 2000–31 December 2018), Finland (1 January 2000–31 December 2016), Iceland (1 January 2004–31 December 2017), Norway (1 January 2005–1 December 2020), and Sweden (1 July 2006–31 December 2019). Each Nordic country maintains national health and social registers^17^ that include information on all births, filled prescriptions (Finland: only filled prescriptions for reimbursed medications in the study material), and diagnoses from inpatient, outpatient specialist care, and those recorded in the medical birth registers (see eAppendix for more detailed description). Reporting to the Nordic registers is mandatory and regulated by national laws.^18^ Individual-level data were deterministically linked based on unique personal identification numbers assigned at birth or immigration. Variable definitions across registers and countries were harmonized through a common data model.^19^

To avoid misclassification of the pregnancy period, we excluded children with missing or invalid data on birth date or gestational age. We also excluded children diagnosed with chromosomal/genetic anomalies or fetal alcohol syndrome. To limit confounding by underlying maternal condition, we restricted the study cohort to include only children born to mothers with psychiatric disorders (eFigure S1).

### Maternal psychiatric disorder

We obtained information on maternal psychiatric disorders from diagnoses recorded in in- and outpatient specialist care, as well as in antenatal care, using International Statistical Classification of Diseases and Related Health Problems, Tenth Revision (ICD-10) codes. We defined maternal psychiatric disorders as any diagnosis of a psychiatric condition (ICD-10 codes: F10–F49, F60–F98, X60–X84, and Y10–Y34) in the 12 months before pregnancy start. For Norway, data from specialist care was available from 2008 only, therefore for births between 2005 and 2010 we also identified psychiatric disorders using reimbursement codes for prescription fills in the 12 months before pregnancy start (eTable S3).

### Antipsychotic exposure

We considered children prenatally exposed to antipsychotics if their mother filled ≥1 prescription for any antipsychotic (Anatomical Therapeutic Chemical (ATC) classification code: N05A, eTable S2) anytime between pregnancy start (based on the date of the first day of the last menstrual period, estimated by using gestational age at birth in days) until the date of birth. We did not consider lithium (ATC code: N05AN01) as an antipsychotic in this study, as it has a different mechanism of action. We classified children as unexposed if their mother did not fill a prescription for any antipsychotic from 90 days before the start of pregnancy until birth. We conducted analyses by timing of antipsychotic exposure: late pregnancy, defined as a prescription fill anytime during the second or third trimesters, and throughout pregnancy, defined as a prescription filled during the first trimester and a prescription filled during late pregnancy (eTable S3). We also assessed exposure by individual antipsychotic monotherapy anytime during pregnancy defined as prescriptions filled for only one type of antipsychotic medication during pregnancy.

### Neurodevelopmental disorders

We ascertained information on child neurodevelopmental disorders from ICD-10 codes recorded in specialist care registers. We considered children to have a disorder of intellectual developmental (ICD-10 codes F70.0–F73.0), developmental speech or language disorder (ICD-10 codes F80.0–F80.2, in the absence of a hearing loss diagnosis), or developmental learning disorder (ICD-10 codes F81.0–F81.9) if they had at least one record of a diagnosis after the age of 3 years or after the age of 5 years for learning disorders. We assessed these diagnoses as non-mutually exclusive, and we defined a composite neurodevelopmental outcome—as having any of the above diagnoses. Similar algorithms have been validated with high positive predictive values to identify diagnoses of neurodevelopmental disorders in claims-based data in the United States^20^ but not validated in the Nordic registers. Hence, we did not include children with an unspecified diagnosis of intellectual developmental. Denmark, due to low numbers, did not contribute results for developmental speech or language- and learning disorders as these diagnoses were not well recorded in settings covered by the National Patient Register but contributed data for the other neurodevelopmental diagnoses.

### Poor academic performance

We assessed poor academic performance in the first national standardized school test administered among school children in mathematics and language arts (i.e., native language skills, including reading and/or writing, and grammar) using national education registers (eAppendix). The grade level at which these tests were first administered differed by country, children were tested between the age of 8 and 10 years (grade levels 2nd to 4th) and in each country the participation rate in these tests exceeded 85%. For this analysis, we restricted to children from countries and calendar years where information on national standardized school tests was available for the study, including children born in Denmark (1 January 2000–31 December 2009), Iceland (1 January 2004–31 December 2007), Norway (1 January 2005–31 December 2010), and Sweden (1 July 2006–31 December 2009) (eFigure S1). We defined poor academic performance as scoring in the lowest 25th percentile, based on standardized scores, or a record of a failure (for children in Sweden). This cut off was chosen to align with the proportion of children in Sweden who failed.

### Covariates

We identified several demographic and clinical covariates a priori as potential (or proxies for) confounders and risk factors for the outcomes defined in eTable S3 (see directed acyclic diagrams eFigures S2 and S3). These covariates were child's country of birth, year of birth and sex, maternal country of birth, education, age, parity, cohabitation, smoking status, and body mass index (BMI) in early pregnancy, other medication use, known or suspected teratogen use, maternal comorbidity during pregnancy, and psychiatric diagnosis group as defined in eTable S3. Maternal education was not available for Finland and for Iceland maternal smoking status in early pregnancy was not available. We imputed missing data for BMI, maternal education, smoking status in early pregnancy, maternal birth country, marital/cohabitation status, and parity using multiple imputation by chained equations (eTable S4).^21^^,^^22^

### Data analysis

In the primary analyses, we estimated the association between the study outcomes and any antipsychotic exposure during pregnancy, timing of antipsychotic exposure, and by most common monotherapies.

We performed multivariable analyses with two levels of adjustment: adjusting minimally for child's country of birth, year of birth, sex of child, maternal age, and parity using outcome regression. In fully adjusted models we applied propensity score overlap weights to balance all maternal baseline covariates and risk factors for outcome including child sex (eTable S3). Overlap weights by definition balances all covariates to a standardized difference of 0, the target population can be interpreted as the population with similar characteristics that could appear with substantial probability in either treatment group.^23^ We estimated separate propensity score for each imputed data set, using logistic regression (eTable S4).

For the neurodevelopmental disorder outcomes, we followed all children from date of birth until the date of outcome event, death, or emigration (not available for Finland), or end of study period, whichever came first. We assessed crude and propensity score-weighted cumulative incidences for each neurodevelopmental disorder separately for the combined cohort and Danish cohort using Kaplan–Meier analyses. We used Cox proportional hazard regression models to calculate adjusted hazard ratios (aHRs) for each neurodevelopmental disorder outcome using the age of the child as the underlying time scale. We included child's country of birth and birth year as strata variables in the models as these variables did not satisfy the proportional hazard assumption.^24^ For the academic performance outcome, we used Poisson regression models to estimate adjusted risk ratios (aRRs) separately for mathematics and language arts. We used robust standard errors to estimate 95% confidence intervals (CI), accounting for multiple births from the same family.

We combined data from Finland, Iceland, Norway, and Sweden and analysed these as one single cohort, while data from Denmark was analysed separately, to comply with national data protection regulations.^19^ We pooled effect estimates using random effects meta-analysis.^25^

### Secondary analyses

In a secondary analysis, we estimated the associations using comparisons which addressed potential sources of unmeasured confounding in the primary analysis. First, we compared the risk of each outcome in children whose mothers were exposed to any antipsychotics during pregnancy to those who discontinued treatment prior to pregnancy i.e., a prescription filled from 12 months to 91 days before pregnancy, but no prescription filled from 90 days to pregnancy. Finland did not contribute to this secondary analysis due to data availability (eAppendix). Second, to address shared genetic and social confounding factors at the family level, we conducted a sibling analysis for each outcome by restricting to siblings discordant for both antipsychotic exposure and outcome.

### Sensitivity analyses

We also conducted several sensitivity analyses to explore the robustness of our findings. First, to reduce potential misclassification of exposure and increase likelihood of that medication was consumed, we re-defined the primary exposure definition as having two or more prescriptions of any antipsychotic filled during pregnancy. Second, to reduce potential outcome misclassification for academic performance, we redefined poor academic performance as scoring in the lowest 10th percentile of their school grade excluding Swedish children for whom this information was not available. Last, to assess whether selection due to non-participation in the national school tests affected the results for academic performance outcomes, inverse probability of selection (censoring) weights was fitted to each model in addition to propensity score overlap weights. To predict the probability of having no test result, we used maternal education, maternal birth country, sex of child and additional covariates including child comorbidity defined by the paediatric comorbidity index,^26^ whether the child had a diagnosis of ADHD and any prescription fill for a psychiatric medication prior to the age of 8 years (eTable S10).

### Post-hoc analyses

For the potential signals of increased risks detected in main analyses for chlorpromazine, we conducted several post-hoc sensitivity analyses to assess the robustness of our findings against unmeasured confounding. We first calculated the E-value to estimate the strength needed for an unmeasured confounder to explain the observed associations.^27^ Second, we repeated the secondary analysis comparing discontinuers with those exposed to chlorpromazine monotherapy and the sensitivity analysis requiring at least two prescription fills for chlorpromazine monotherapy.

We conducted all analyses in R (version 4.2.1). This study followed the REporting of studies Conducted using Observational Routinely collected health Data (RECORD) reporting guidelines for observational studies (eTable S12).

### Ethics statement

In the Nordic countries, register-based studies are either exempt from the requirement to obtain informed consent from individuals or need to be granted a waiver of the need for informed consent for the specific research project. The relevant ethical and/or data protection authorities in all study countries approved the project and granted a waiver of informed consent, where appropriate (eTable S1).

### Role of the funding source

The funders of the study had no role in study design, data collection, data analysis, data interpretation, the writing of the report or the decision to submit the manuscript for publication.

## Results

### Cohort characteristics

In a source population of 4,431,872 million children, we identified 212,342 children born to women with a recorded psychiatric diagnosis and eligible to be included in the study cohort. Overall, 11,626 children (5.5%) were prenatally exposed to antipsychotics; thereof 2443 (21%) in the second or third trimester only, and 4801 (41%) throughout the pregnancy period. Quetiapine (n = 4492), olanzapine (n = 1400), prochlorperazine (n = 716), perphenazine (n = 661), aripiprazole (n = 523), and levomepromazine (n = 380) were the most common monotherapies used during pregnancy. Among the sub-cohort of children with available academic data (n = 40,969), 1958 (4.8%) were exposed to antipsychotics prenatally. Women treated with antipsychotics during pregnancy were more likely to be older (35 years or more), have a lower education level and a higher BMI, to smoke, and have greater use of other medications during pregnancy, compared with those not exposed to antipsychotics during pregnancy (Table 1). After propensity score weighting, all covariates were perfectly balanced between exposed and unexposed groups to have a standardized mean difference of 0, as per definition of overlap weights.

**Table 1:** Maternal and child characteristics by exposure to antipsychotics during pregnancy, for births between 2000 and 2020.

Maternal characteristics	Finnish, Icelandic, Norwegian, and Swedish cohort			Danish cohort		
	Unexposed (n = 163,604)	Exposed (n = 9676)	SMD	Unexposed (n = 33,692)	Exposed (n = 1950)	SMD
	n (%)	n (%)		n (%)	n (%)	
**Age at delivery, years**						
<20	7001 (4.3%)	319 (3.3%)	0.098	1450 (4.3%)	53 (2.7%)	0.175
20–24	29,176 (17.8%)	1687 (17.4%)		6911 (20.5%)	384 (19.7%)	
25–29	46,325 (28.3%)	2657 (27.5%)		10,032 (29.8%)	541 (27.7%)	
30–34	47,437 (29.0%)	2715 (28.1%)		9356 (27.8%)	511 (26.2%)	
35–39	26,857 (16.4%)	1744 (18.0%)		4881 (14.5%)	355 (18.2%)	
≥40	6808 (4.2%)	554 (5.7%)		1062 (3.2%)	106 (5.4%)	
**Education**^a^						
Compulsory	34,496 (21.1%)	2157 (22.3%)	0.24	13,185 (39.1%)	1013 (51.9%)	0.28
Secondary	55,228 (33.8%)	2469 (25.5%)		12,376 (36.7%)	616 (31.6%)	
Post-secondary	43,006 (26.3%)	1435 (14.8%)		7634 (22.7%)	291 (14.9%)	
*Missing*	30,874 (18.9%)	3615 (37.4%)		497 (1.5%)	30 (1.5%)	
**Cohabiting**						
Yes	133,407 (81.5%)	7203 (74.4%)	0.17	13,560 (40.2%)^b^	766 (39.3%)	0.02
No	29,345 (17.9%)	2402 (24.8%)		20,132 (59.8%)	1184 (60.7%)	
*Missing*	852 (0.5%)	71 (0.7%)		–	–	
**Maternal birth country**						
Within country of delivery	136,564 (83.5%)	7837 (81.0%)	0.007	29,003 (86.1%)	1537 (78.8%)	0.192
Outside country of delivery	22,368 (13.7%)	1311 (13.5%)		4689 (13.9%)	413 (21.2%)	
*Missing*	4672 (2.9%)	528 (5.5%)				
**BMI, early pregnancy**						
<18.5	4980 (3.0%)	269 (2.8%)	0.202	1796 (5.3%)	89 (4.6%)	0.213
18.5–24	70,559 (43.1%)	3395 (35.1%)		14,703 (43.6%)	678 (34.8%)	
25–29	32,382 (19.8%)	2154 (22.3%)		6785 (20.1%)	450 (23.1%)	
≥30	21,740 (13.3%)	1849 (19.1%)		5057 (15.0%)	407 (20.9%)	
*Missing*	33,943 (20.7%)	2009 (20.8%)		5351 (15.9%)	326 (16.7%)	
**Smoking, early pregnancy**^c^						
Yes	30,651 (18.7%)	2998 (31.0%)	0.314	11,933 (35.4%)	857 (43.9%)	0.191
No	120,104 (73.4%)	5780 (59.7%)		21,004 (62.3%)	1024 (52.5%)	
*Missing*	12,849 (7.9%)	898 (9.3%)		755 (2.2%)	69 (3.5%)	
**Parity**						
0	75,478 (46.1%)	4795 (49.6%)	0.107	16,822 (49.9%)	1005 (51.5%)	0.159
1	52,980 (32.4%)	2648 (27.4%)		10,458 (31.0%)	482 (24.7%)	
≥2	34,484 (21.1%)	2148 (22.2%)		6412 (19.0%)	463 (23.7%)	
Missing	662 (0.4%)	85 (0.9%)				
**Maternal comorbidity**						
Yes	11,507 (7.0%)	846 (8.7%)	0.063	1513 (4.5%)	124 (6.4%)	0.083
**Maternal psychiatric diagnosis**						
Psychotic or bipolar disorders	9275 (5.7%)	4186 (43.3%)	0.996	1695 (5.0%)	863 (44.3%)	1.022
Other psychiatric disorders	146,253 (89.4%)	4897 (50.6%)		31,997 (95.0%)	1087 (55.7%)	
Psychiatric disorders recorded by prescription reimbursement^d^	8076 (4.9%)	593 (6.1%)		–	–	
**Use of known/suspected teratogens during pregnancy**						
Yes	15,039 (9.2%)	2713 (28.0%)	0.499	2277 (6.8%)	513 (26.3%)	0.545
**Other medications during pregnancy**						
Yes	81,801 (50.0%)	7104 (73.4%)	0.496	14,911 (44.3%)	1434 (73.5%)	0.623
**Sex of child**						
Female	79,411 (48.5%)	4791 (49.5%)	0.02	16,272 (48.3%)	925 (47.4%)	0.017
Male	84,193 (51.5%)	4885 (50.5%)		17,420 (51.7%)	1025 (52.6%)	
**Child calendar year of birth**						
2000–2005	8655 (5.3%)	616 (6.4%)	0.06	6742 (20.0%)	363 (18.6%)	0.036
2006–2011	54,335 (33.2%)	3203 (33.1%)		11,985 (35.6%)	699 (35.8%)	
2012–2017	74,250 (45.4%)	4448 (46.0%)		14,965 (44.4%)	888 (45.5%)	
2018–2020	26,364 (16.1%)	1409 (14.6%)		–	–	

ADHD, attention-deficit/hyperactive disorder; BMI, body mass index; SMD, standardised mean difference.

a Not available in Finland.

b In Finland, Iceland, Norway and Sweden, cohabitating refers to any situation where the mother reports living with a partner, in Denmark cohabiting refers to married or registered partnerships only.

c Not available in Iceland.

d Psychiatric disorders recorded as the indication for prescription using reimbursement codes for chronic psychiatric disorders for women in Norway with births between 2005 and 2010.

### Risk of neurodevelopmental disorders

**Fig. 1:**
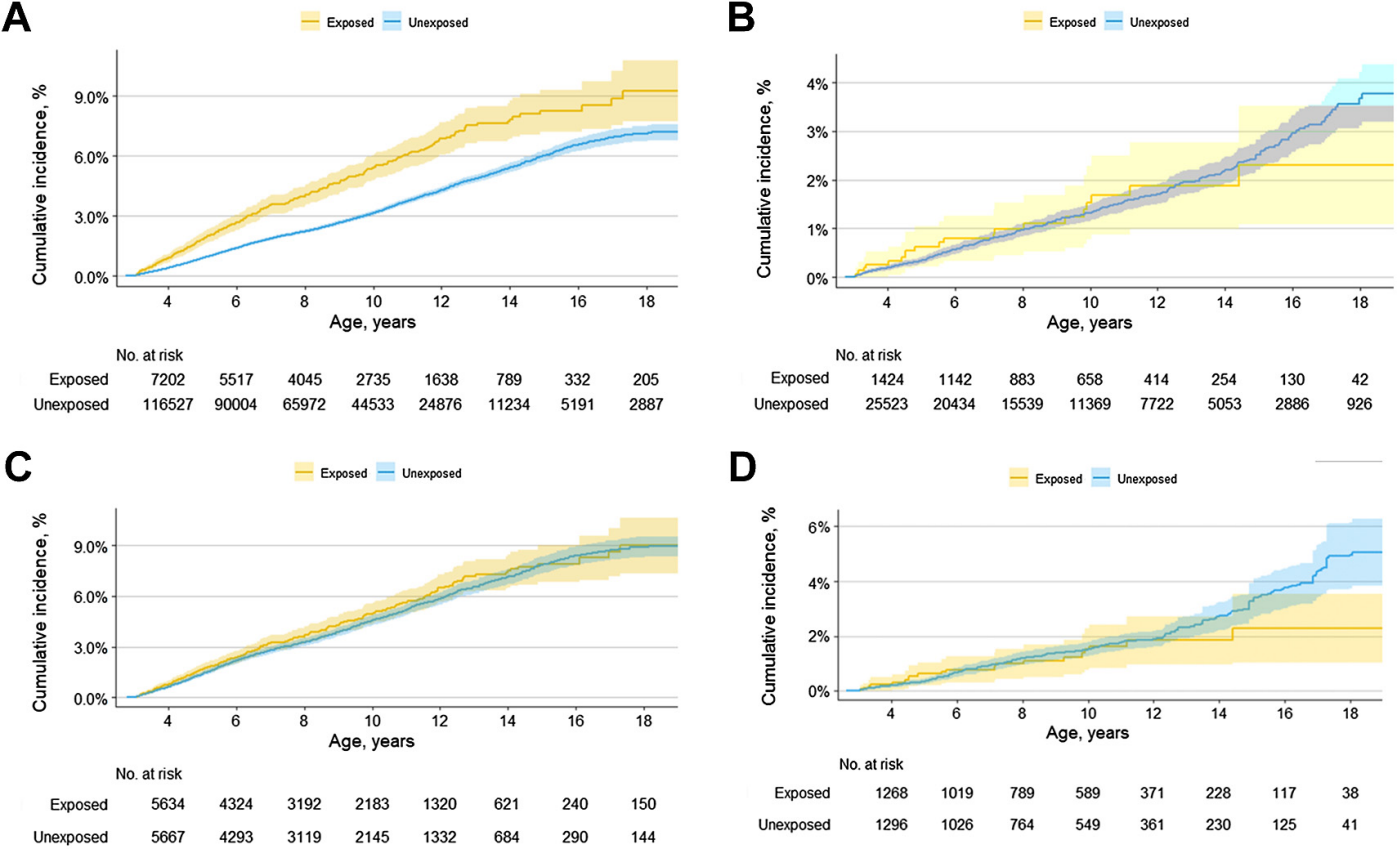
Crude and propensity score-weighted cumulative incidence (with 95% confidence intervals) for composite neurodevelopmental outcome from age 3–19 years in children of mothers with psychiatric disorders born between 2000 and 2020 by prenatal antipsychotic exposure. A) Crude cumulative incidence (with 95% confidence intervals) in combined cohort (Finland, Iceland, Norway, Sweden) B) Crude cumulative incidence (with 95% confidence intervals) in Denmark C) Propensity score-weighted cumulative incidence (with 95% confidence intervals) in combined cohort (Finland, Iceland, Norway, Sweden) D) Propensity score-weighted cumulative incidence (with 95% confidence intervals) in Denmark. ^a^Adjusted for birth year, sex of child, child’s country of birth, maternal birth country, age, parity, education, cohabitation status, BMI & smoking in early pregnancy, use of other medications during pregnancy, or known/suspected teratogens and comorbidity prior to pregnancy using propensity score overlap weights.

**Fig. 2:**
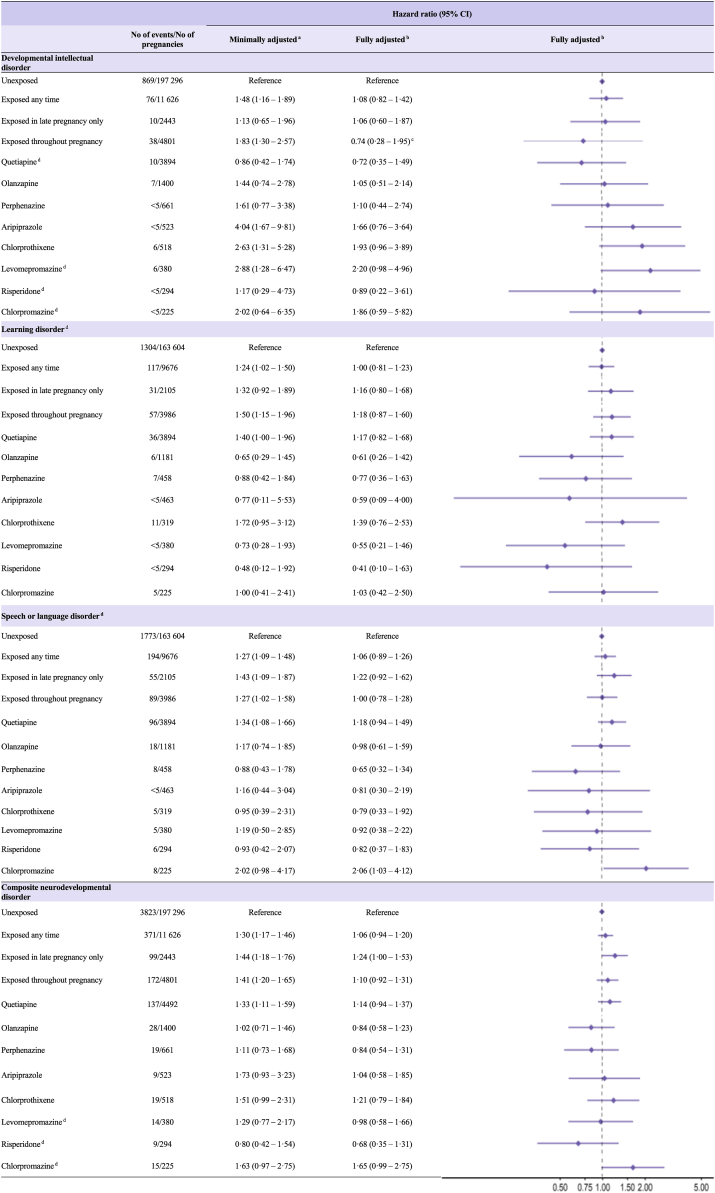
Hazard ratios (with 95% confidence intervals) for child neurodevelopmental disorders after prenatal antipsychotic exposure by timing of exposure and monotherapy. BMI, body mass index; CI, confidence interval Monotherapy was assumed if a prescription was filled for only one type of antipsychotic medication during the exposure period. For numbers less than 5 (<5), numbers are suppressed due to country-/data-specific suppression policy. ^a^Adjusted for birth year and sex of child, child's country of birth, maternal age, parity using outcome regression. ^b^Adjusted for birth year, sex of child, child's country of birth, maternal age, parity, education, maternal birth country, cohabitation status, BMI & smoking in early pregnancy, use of other medications during pregnancy, or known/suspected teratogens and comorbidity prior to pregnancy using propensity score overlap weights. ^c^Substantially different estimates were observed from Denmark versus the combined Nordic data (*I^2^* = 92%). Denmark, HR 0.35 (95% CI 0.09–0.64); combined Nordic cohort, 1.44 (0.94–2.20). ^d^Results presented only for the combined cohort (Finland, Iceland, Norway, and Sweden), owing to low number of exposure/outcomes in the Danish cohort.

The crude cumulative incidence of composite neurodevelopmental disorders, diagnosed by the age of 8 years in the combined cohort, was 4.0% (95% confidence interval 3.5%–4.5%) for children with prenatal exposure to antipsychotics compared with 2.2% (2.1%–2.3%) in unexposed children (Fig. 1, see eTable S5 for overall incidences). Risk estimates for neurodevelopmental disorders attenuated in fully adjusted models, and the aHRs did not suggest an increased risk for most exposure–outcome associations (Fig. 2). However, we observed a slightly positive association for any neurodevelopmental disorder with antipsychotic exposure during late pregnancy, 1.24 (95% CI 1.00–1.53). Both the minimally and fully adjusted HRs were elevated with chlorpromazine monotherapy for the composite neurodevelopmental outcome, 1.65 (0.99–2.75), and specifically for speech or language disorders 2.06 (1.03–4.12), but the number of exposed events were low (n = 15, n = 8, respectively).

### Risk of poor academic performance

**Fig. 3:**
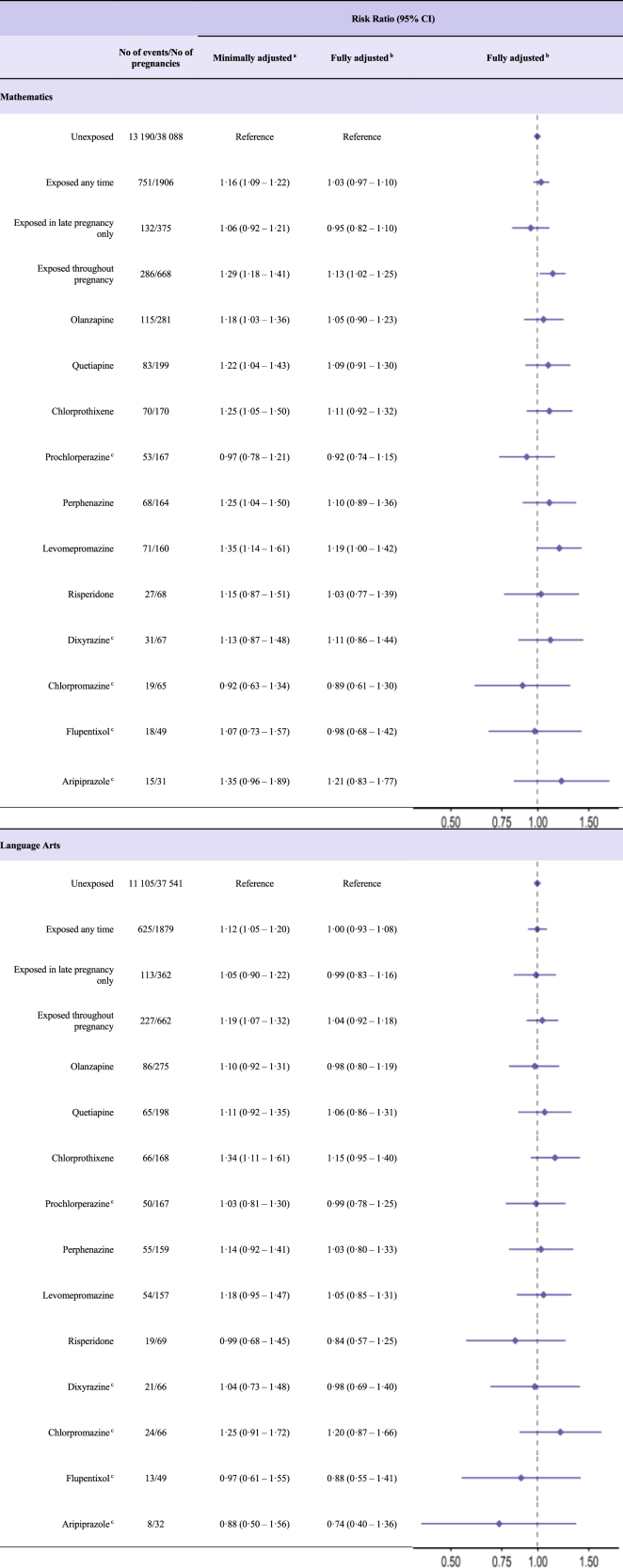
Risk ratios (with 95% confidence intervals) for poor academic performance after prenatal antipsychotic exposure by timing of exposure and monotherapy. BMI, body mass index; CI, confidence interval. Monotherapy was assumed if a prescription was filled for only one type of antipsychotic medication during the exposure period. For numbers less than 5 (<5), numbers are suppressed due – country-/data-specific suppression policy. Finland was not included in this analysis due to data availability. ^a^Adjusted for birth year and sex of child, child’s country of birth, maternal age, parity using outcome regression. ^b^Adjusted for birth year, sex of child, child’s country of birth, maternal age, parity, education, maternal birth country, cohabitation status, BMI and smoking in early pregnancy, use of other medications during pregnancy, or known/suspected teratogens and comorbidity prior to pregnancy using propensity score overlap weights. ^c^Results presented only for the combined academic cohort (Iceland, Norway, and Sweden), owing to low number of exposure/outcomes in the Danish cohort.

Among children with available academic test scores, 34,643 had test results in mathematics and 34,351 in language arts. Prenatal exposure to antipsychotics was not associated with poor academic performance in mathematics or language arts, with fully adjusted RRs of 1.04 (95% CI 0.91–1.18) and 1.00 (0.87–1.15), respectively (Fig. 3). The risk estimates for poor academic performance remained similar across different timing of exposure in pregnancy. Examining individual antipsychotics as monotherapies, only levomepromazine was associated with a marginally elevated risk of poor performance in mathematics (aRR 1.19, 1.00–1.42) but not in language arts (aRR 1.05, 0.85–1.31).

### Secondary analyses

**Fig. 4:**
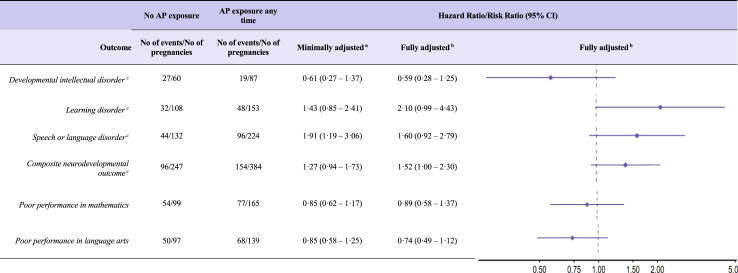
Hazard and risk ratios (with 95% confidence intervals) for child neurodevelopmental disorders and poor academic performance after prenatal antipsychotic exposure in sibling matched analysis where siblings are discordant on exposure and outcome. AP, antipsychotic; BMI, body mass index; CI, confidence interval; *I^2^, I²* statistic ^a^ Adjusted for birth year and sex of child, child’s country of birth, maternal age, parity using outcome regression. ^b^ Adjusted for birth year, sex of child, child’s country of birth, maternal birth country, age, parity, education, cohabitation status, BMI & smoking in early pregnancy, use of other medications during pregnancy, or known/suspected teratogens and comorbidity prior to pregnancy using propensity score overlap weights. ^c^ Results presented only for the combined cohort from Finland, Iceland, Norway, and Sweden, owing to low number of exposure/outcomes in the Danish cohort.

Comparing children whose mothers used antipsychotics during pregnancy with those whose mothers’ discontinued treatment before pregnancy showed no increased risks for any measured neurodevelopmental outcome nor for poor academic performance (eTable S6). Restricting the analysis to siblings discordant for both exposure and outcome did not yield any clear associations for neurodevelopmental disorders or poor academic performance (Fig. 4). However, when examining the risk of the composite neurodevelopmental outcome in the fully adjusted sibling model the HR from the combined Nordic data was 1.52 (1.00–2.30).

### Sensitivity analyses

The sensitivity analyses yielded results largely consistent with those observed in the primary analysis.

Exposure misclassification: All estimates remained similar when defining prenatal exposure as at least two filled antipsychotic prescriptions (eTable S7).

Outcome misclassification: We also observed similar findings when poor academic performance was re-defined as the lowest 10th percentile of scores (eTable S8).

Selection bias due to non-participation in standardized academic tests: Among a total of 45,752 children born in the relevant countries and covered by years with available test data, we identified 4783 (10%) non-participants (8% in combined cohort, 18% in Danish cohort). Non-participating children had higher scores in the paediatric comorbidity index, and they were more likely to be born to mothers who smoked during early pregnancy, did not cohabit with a partner, had a low educational level, and/or were of foreign origin. Notably, maternal psychiatric disorders and exposure to antipsychotics did not differ between children who had a test result and those who did not, standardized mean difference <0.01 (eTable S9). When accounting for selection bias using censoring weights, most results did not differ from findings of the main analysis (eTable S10).

### Post-hoc analyses

The E-score indicated that an unmeasured confounder would need to have an association with effect size of 3.5 with both chlorpromazine exposure and speech and language disorders (or 2.6 for the composite outcome) after adjusting for all measured confounders to explain away the observed signal among chlorpromazine exposed. An unmeasured confounder with an adjusted effect size of 1.2 with both exposure and outcome would shift the confidence interval to include 1. We observed positive associations when comparing chlorpromazine user with discontinuers for composite outcome (aHR 2.17, 95% CI 1.09–4.35) (eTable S11), but no associations when requiring at least two prescription fills for chlorpromazine monotherapy compared to unexposed (aHR 1.01, 0.36–2.83).

## Discussion

In this large population-based cohort study of children born to women with psychiatric disorders, we did not find evidence that prenatal antipsychotic exposure increases the risk of child neurodevelopmental disorders or learning difficulties. After accounting for measured confounders, risk estimates attenuated for all antipsychotics except chlorpromazine. Findings were consistent across a range of pre-specified secondary and sensitivity analyses.

We expanded considerably on the existing evidence on long-term safety of antipsychotics by combining data from multiple countries with similar health and education systems. The large source population allowed us to examine individual antipsychotic medications and control for confounding by maternal indication by restricting our study population to children of women with a diagnosed psychiatric disorder. Nevertheless, disentangling the potential effects of antipsychotics from the underlying maternal illness and associated factors remains challenging. But the even balance of covariates between exposed and unexposed children achieved through propensity score weighting, together with the clear attenuation of risk towards the null for all outcomes in fully-vs. minimally adjusted models, suggest that we were largely able to overcome measured confounding. Requiring a recorded diagnosis may mean our results are more generalisable to children born to women with moderate to severe psychiatric illnesses who are more closely followed in specialist health care services.

Our findings are largely consistent with a recent health insurance claims-based study in the United States examining developmental intellectual, learning and speech or language disorders, along with other neurodevelopmental disorders, after prenatal antipsychotic exposure.^13^ They also align with our previous findings of no association with ADHD and autism spectrum disorder after exposure during pregnancy, which were based on a similar approach and underlying data.^14^ Our study is the first population-based cohort study to provide evidence on the effect of chlorpromazine and long-term neurodevelopment, making it difficult to make comparisons to the signal detected in our data. Prenatal chlorpromazine exposure has been associated with transient effects on cognitive-, motor-, social-emotional and adaptive skills in children in their first year of life.^12^ Animal studies have also suggested motor impairment and delays in reaching developmental milestones following prenatal exposure to chlorpromazine.^12^ While we observed positive associations between chlorpromazine and speech or language disorders, the estimate was limited by small sample size and not consistent across other outcomes. Our post-hoc sensitivity analyses indicate that unmeasured confounding may only explain the elevated effect estimates to a small degree, thus a chance finding due to small numbers and multiple testing is plausible. Chlorpromazine is a medication with diminishing use in pregnancy across several countries^7^ and is no longer on the Nordic market. Regardless, where possible patients and clinicians may choose alternative antipsychotics to chlorpromazine.

Poor academic performance can serve as a useful marker of cognitive functioning, as well as an indicator for subclinical developmental impairment.^28^ Our study findings regarding academic performance on national standardized tests are consistent with recent null findings reported by a population-based study in Denmark.^16^ Liu et al.^16^ also reported null findings for prenatal exposure and poor performance when stratifying on maternal psychiatric disorders including psychotic disorders, mood disorders, neurotic, stress-related and somatoform disorders, and no diagnosed psychiatric disorders, but a positive association in the small group of mothers with ‘other’ psychiatric disorders. Our findings corroborated those of a clinical study^29^ which examined IQ and neurodevelopmental functioning using a range of detailed cognitive assessments in a small sample of children of mothers with psychiatric conditions. Robust confounding control is necessary when examining school performance, as genetic variants associated with increased risk for psychiatric disorders, particularly schizophrenia are also associated with poor performance in mathematics and/or better performance in language arts.^30^

Our study had some important limitations. First, we used specialist outpatient and inpatient records which may not capture all children with learning and speech or language disorders as these may be managed outside these institutions. Second, not all children participated in school tests, which may have led to selection bias. However, we were able to use a range of covariate information on all eligible children to apply inverse censoring weighting to account for potential differences among non-participating children and this yielded similar results as our main analysis. Third, mothers who filled an antipsychotic prescription during pregnancy may not have taken their medication, which would lead to exposure misclassification. To address this limitation, we conducted a sensitivity analysis requiring two prescription fills during pregnancy, the findings of which corroborated our main analysis. Fourth, the cohort was restricted to live births which could theoretically have resulted in underestimation of risks due survivorship bias if prenatal antipsychotic exposure was strongly associated with spontaneous and induced abortion or stillbirth, independently from the underlying maternal psychiatric condition. Fifth, although we accounted for confounding by lifestyle using proxies such as BMI and smoking during pregnancy and through sibling analyses, we cannot rule out residual confounding due to unmeasured confounders including those occurring after birth such as ongoing mental illness prior to academic testing. Our estimates would likely have been even closer to the null if we had been able to completely control for lifestyle factors. Lastly, for some medications, low exposure and outcome numbers hampered our ability to draw firm conclusions. Still, we chose to include these for completeness and transparency and to facilitate their inclusion in future meta-analyses.

This large multi-national cohort study found that the most commonly used antipsychotics during pregnancy, including quetiapine and olanzapine, did not increase risk of neurodevelopmental disorders or poor academic performance among the children of women with psychiatric disorders. Our findings provide robust evidence with respect to long-term safety of antipsychotic treatment, which may assist and reassure clinicians and women managing mental illness during pregnancy or planning pregnancy.

## Contributors

HZ, JMC, CEC and CB contributed to all the aspects of this study. ØK, MKL, VH, and SPU were involved in preparation of analytic datasets, designing the study, and preparation of the final manuscript. MHB, BOE, MBG, MG, AH, MN, SP, JR, KF were involved in designing the study and preparation of the final manuscript. HZ, JMC, CB, CEC, MKL, VH, and KF had access to data or statistical reports and tables from Finland, Iceland, Norway, and Sweden. MN, BOE, and SPU had access to the Danish data. All authors with access to data take responsibility for the integrity of the data and the accuracy of the data analysis. HZ, JMC, and CB are the guarantors. All authors contributed important intellectual insights to the critical revision of the manuscript and approved its final version. The corresponding author attests that all listed authors meet authorship criteria and that no others meeting the criteria have been omitted.

## Data sharing statement

The data used for this study from national health and social registers are available to other researchers upon ethical approval and application to the register holders. The authors may not share the study data due to regulations which restrict access and distribution to those with ethical and legal permission to use the data.

## Declaration of interests

CEC and JR are employees of the Centre for Pharmacoepidemiology at Karolinska Institutet, which receives funding from several entities (pharmaceutical companies, regulatory authorities, contract research organizations) for the performance of drug safety and drug utilization studies, unrelated to this work. KF, ØK, and VH report participation in regulator mandated phase IV studies (PASS) unrelated to the submitted work, funded by pharmaceutical companies (Novo Nordisk, LEO Pharma and Bristol Myers Squibb) and paid to the institution (no personal fees). MG and MKL Leinonen report a grant from the Innovative Medicines Initiative (IMI ConcePTION, grant agreement number 821520) while conducting the study, unrelated to this work. MG and MKL also report that their institution has received funding from pharmaceutical companies to conduct regulator mandated post-marketing drug safety research outside the submitted work. MHB reported fees paid to her institution by valproate market authorization holders for EMA-mandated contract research (PASS studies); speaking and/or consultancy honoraria from Eisai, Novartis Norway, Jazz Pharmaceuticals, Angelini Pharma, AbbVie, Teva, Lilly, and Lundbeck unrelated to the medications in the study. All other authors do not report any competing interests.

## Appendix A Supplementary data

Supplmentary material
