# Supplementary material for: Antipsychotic use during pregnancy and risk of specific neurodevelopmental disorders and learning difficulties in children: a multinational cohort study
Source: eClinicalMedicine. 2024 Mar 17;70:102531. doi: 10.1016/j.eclinm.2024.102531 (PMC11056394; doi:10.1016/j.eclinm.2024.102531)
Supplement: Supplmentary material [file mmc1.docx]

**Supplementary file**

[eAppendix. Detailed descriptions of registers used in this study. 2](#_Toc158883346)

[eFigure S1. Flowchart study cohort derived from Finland, Iceland, Norway, and Sweden (combined cohort) and Denmark (Danish cohort). 5](#_Toc158883347)

[eFigure S2. Directed Acyclic Graph of possible causal pathways relevant to prenatal antipsychotic exposure and risk of child neurodevelopmental disorders. 6](#_Toc158883348)

[eFigure S3. Directed Acyclic Graph of possible causal pathways relevant to prenatal antipsychotic exposure and risk of poor school performance A) with potential selection bias and B) addressing selection bias with inverse probability censor weights. 7](#_Toc158883349)

[eTable S1. Ethical boards providing approval for data use & approval numbers. 8](#_Toc158883350)

[eTable S2. Anatomical Therapeutic Chemical classification codes for antipsychotic medications included in study. 9](#_Toc158883351)

[eTable S3. Exposure and covariate definitions, source data, and relevant diagnostic, medication codes. 10](#_Toc158883352)

[eTable S4. Multiple imputations by chained equations. 13](#_Toc158883353)

[eTable S5. Crude cumulative incidence (with 95% confidence intervals) for each neurodevelopmental disorder in children of mothers with psychiatric disorders. 14](#_Toc158883354)

[eTable S6. Secondary analysis: hazard ratios and risk ratios (with 95% confidence intervals) for child neurodevelopmental disorders and poor academic performance when comparing children prenatal exposed with children whose mothers discontinued prior to pregnancy. 15](#_Toc158883355)

[eTable S7. Sensitivity analysis: hazard ratios and risk ratios (with 95% confidence intervals) for child neurodevelopmental disorder and poor academic performance when redefining exposure as at least two prescription fills any time during pregnancy. 16](#_Toc158883356)

[eTable S8. Sensitivity analysis: risk ratios (with 95% confidence intervals) for poor academic performance after prenatal antipsychotic exposure by timing of exposure when redefining poor performance outcome as scoring in the lowest 10th percentile. 17](#_Toc158883357)

[eTable S9. Comparison of maternal and child characteristics for children with and without a national standardised test result born between 2000 and 2011. 18](#_Toc158883358)

[eTable S10. Inverse probability of selection (censoring) analysis: risk ratios (with 95% confidence intervals) for poor academic performance after prenatal antipsychotic exposure by timing of exposure and monotherapy. 20](#_Toc158883359)

[eTable S11. Post-hoc analysis: hazard ratios (with 95% confidence intervals) for child neurodevelopmental disorders when comparing prenatal chlorpromazine monotherapy exposure to women who discontinued antipsychotic treatment before pregnancy. 23](#_Toc158883360)

[eTable S12. REporting of studies Conducted using Observational Routinely collected health Data (RECORD) reporting guidelines for observational studies.^9^ 24](#_Toc158883361)

[eReferences 27](#_Toc158883362)

eAppendix. Detailed descriptions of registers used in this study.

Data on **births, filled or reimbursed prescriptions, academic performance, and covariates** were obtained from the medical birth registries of Denmark, Iceland, Norway, Finland, and Sweden linked with prescription, academic performance, death, specialist/hospital care, and population registers which document migration and educational attainment of the population. ^1^

Denmark

The **Danish Medical Birth Registry** was established in 1973 based on paper records and has been entirely electronic since 1995. The registry comprises data on all live births and stillbirths born to mothers with permanent residence in Denmark. Stillbirth was defined as pregnancy loss from 28 complete gestational weeks until April 2004. After this period, the pregnancy loss after 22 complete weeks was considered a stillbirth. The database includes birth characteristics: gestational age, birth weight, Apgar score at 5 minutes, parity, the date of birth, sex, singleton or not, maternal smoking during pregnancy. Data on age and personal identification of mother and child are of good quality. ^2^

The **Danish National Prescription Registry** contains information on all prescriptions dispensed at community pharmacies in Denmark since 1995. The registry started in 1994, but with incomplete registration in the first year. The registry includes variables related to drug user, dispensing (Anatomical Therapeutic Chemical (ATC) classification code, date of dispensing, dose unit and dosage, etc.), prescriber and pharmacy. ^3^

The **National Patient Register** contains dates of hospitalizations and visits, all diagnoses (International Statistical Classification of Diseases and Related Health Problems (ICD) codes) and surgical procedures (NOMESCO Classification of Surgical Procedures (NCSP) codes) from inpatient stays, outpatient specialist care, and emergency room visits since 1977. ^4^

The **Danish Agency for IT and Learning** provided standardised test results for children participating in yearly national testing program called The National Tests. From 2010, the program consisted of 10 mandatory tests: reading tests (grades 2, 4, 6, and 8), math tests in grades 3 and 6, and other tests on different topics (geography, physics, chemistry, and biology) in grades 7 and 8.

Finland

The **Finnish Medical Birth Register:** Since 1987, mandatory registration of all live births and stillbirths of at least 500 grams or 22 completed gestational weeks, as well as data on mothers.

**Register of Reimbursed Drug Purchases and Register of Medical Special Reimbursements**. Nationwide databases on reimbursed drug purchases available from 1995. If an individual is entitled to special reimbursement, then drug refill is accompanied with indication for which the prescription was reimbursed. Prescription data was only provided from 90 days before last menstrual period until birth for each pregnancy.

The **Finnish Patient Registers:** **Care Register for Health Care** contains dates of hospitalizations and visits, all diagnoses (ICD codes) and surgical procedures (NCSP codes) from inpatient stays and outpatient specialist care since 1967/1998.

**Finnish Register of Congenital Malformations** contains all major congenital anomalies (including terminations and stillbirths) identified within one year of birth and some thereafter, which have been validated against medical records.

Iceland

**Icelandic Medical Birth Registry**: Since 1981, mandatory registration of all live- and stillbirths of at least 500 grams or 22 completed gestational weeks.

**Icelandic Prescription Medicines Register**: Since 2003, mandatory registration of all outpatient drugs dispensed in Iceland. Prescription drugs administered to individuals in nursing homes are included after from 2011 onwards.

**Icelandic Patient Register** contains dates of hospitalizations and visits, all diagnoses (ICD-10 codes) and surgical procedures (NCSP codes) from inpatient stays since 1999. An adjuvant register contains outpatient diagnoses from primary care visits from 2005.

**State Diagnostic and Counselling Centre**is a national institution serving children with developmental disabilities and their families. The database contains specialist diagnoses (ICD-10 codes) from all referrals to the Centre with suspicion of autism spectrum disorders, intellectual disorders, and motor disorders.

**Centre for Child Development and Behavior**is a national institution serving children from the whole country with neurodevelopmental, behavioural, and emotional problems and disorders (although it belongs to the Primary Health Care of the Capital Area). The database contains specialist diagnoses of these disorders (ICD-10 codes).

**Icelandic Cause of Death Registry**is a centralised national registry that is maintained by the Directorate of Health and contains mortality data for the Icelandic population, categorised according to the ICD-10 classification system. This includes data on date of death and the main underlying cause of death for each deceased individual.

**Statistics Iceland**: Migration (immigration/emigration), educational attainment.

**Icelandic Directorate of Education** is a governmental institution that provides educational material to students of primary and upper secondary schools in Iceland, supervises the educational system, and conducts standardised testing. The database contains results from Icelandic National Examinations conducted every year for children in 4^th^, 7^th^ and 9^th^ grade since the 1980s.

Norway

**Medical Birth Registry of Norway:** Since 1967, mandatory registration of all live- and stillbirths from 16 completed gestational weeks (12 weeks from 2002, however miscarriages below 16 weeks are incomplete). Includes information about maternal health, pregnancy and delivery complications, and health of the infant.

**Norwegian Prescription Database:** Since January 2004, mandatory registration of all drugs dispensed in Norway, with a code identifying the indication for reimbursement for chronic conditions. The database includes variables related to drug user, dispensing (ATC classification code, date of dispensing, dose unit and dosage, etc.), prescriber and pharmacy. Drugs administered in hospitals or nursing homes with no individual prescription and drugs sold over-the-counter are not included.

**Norwegian Patient Registry:**Dates, diagnoses and procedures from specialists and inpatient care since 2008.

**Norwegian Cause of Death Registry:**Contains data since 1951 on date of death.

**Norwegian Population Register:** Migration/moving, marriage and cohabitation.

**National Education Database:**Established in 1970 and updated every year, the database contains information about the level of education of every Norwegian inhabitant who has received education in Norway from the age of 16 years. Since 2007 (2007/2008 academic year) it contains the results of national tests in primary school and information about whether a pupil participated or was provided an exemption.

Sweden

**The** **Swedish Medical Birth Register** covers since 1973, 99% of all livebirths and stillbirths from gestational week 28. Since July 1, 2008 stillbirths occurring from week 22 are also included. Data is collected from antenatal care clinics, delivery units and pediatric examination of the infant.

**The Swedish** **Prescribed Drug Register** was established in July 2005 and contains complete national data on all pharmaceuticals dispensed on prescription.  Drugs are recorded according to the ATC classification system, with the date of dispensation, the strength, and quantity. Drugs administered in hospitals or nursing homes with no individual prescription and drugs sold over-the-counter are not included.

**The** **Swedish Patient Register** contains dates of hospitalizations and visits, all diagnoses (ICD codes) and surgical procedures (NCSP codes) from inpatient stays. The reporting started in 1964 and since 1987, the register has covered all public, inpatient care in Sweden. Since 2001 both public and private care is included in the register and since 2002 there is almost full coverage of all outpatient hospital visits.

The **National School Register** contains data on students and their school grades and test results collected by Swedish National Agency for Education (Skolverket) and distributed to researchers by Statistics Sweden. In Sweden, children start school in the fall of the year of their seventh birthday. The education of children up to adolescence is compulsory, and primary school is 9 years in total. In the third, sixth and ninth grade, all students do national tests. The national tests in the third grade started in 2010. The national tests at third grade contain seven or eight subtests in Mathematics and Swedish. To receive a passing result, all the subtests within the subject are required.


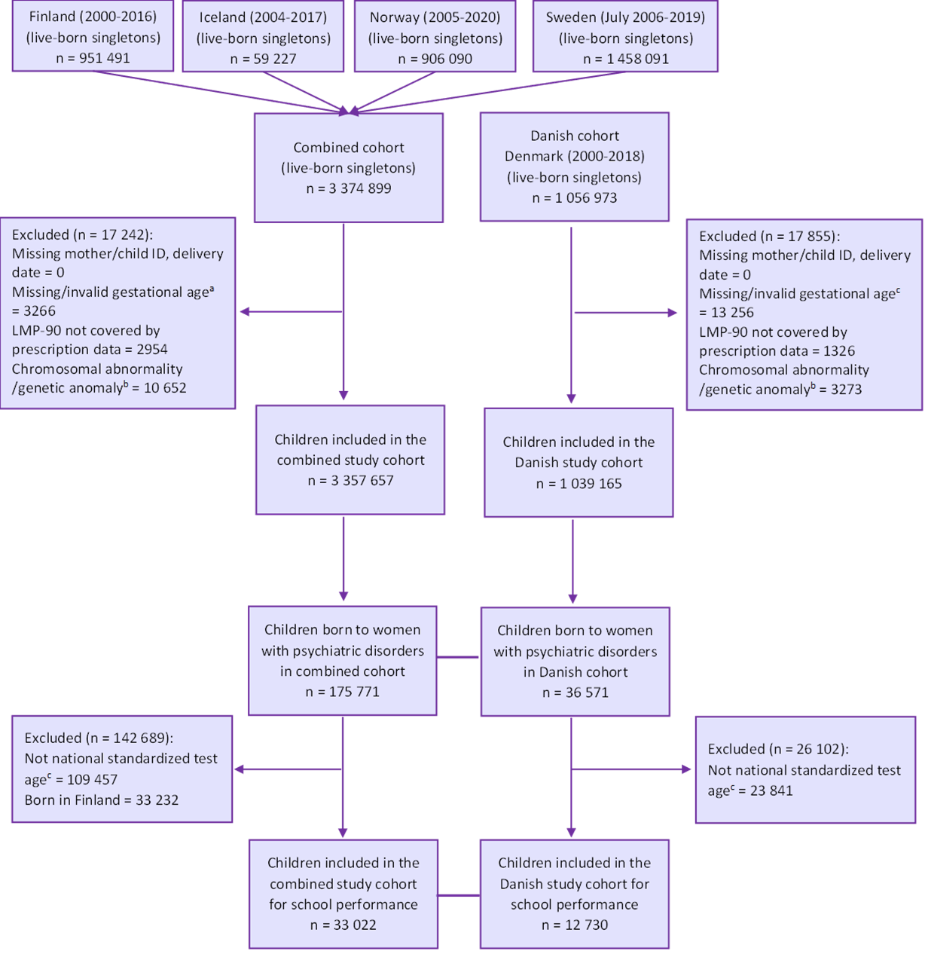
eFigure S1. Flowchart study cohort derived from Finland, Iceland, Norway, and Sweden (combined cohort) and Denmark (Danish cohort).

ICD, International Statistical Classification of Diseases and Related Health Problems; ID, identifier; LMP-90, 90 days before last menstrual period; MBR, Medical birth register; NPR, National patient register

^a^ Criteria for exclusion: excluded if gestational age missing or gestational days ≤154 or >308.

^b^ Criteria for exclusion: excluded if had at least 1 recorded diagnosis of a chromosomal abnormality/genetic anomaly recorded any time in the NPR, MBR, or register of congenital anomalies of the following:
ICD-10 codes: D821, P350, P351, P371, Q751, Q754, Q87, Q90 – Q94, Q96–Q99, Q860 &
ICD-9 Atlanta codes: 27911, 75581, 75604, 7580-7583, 7585-7589, 75980-75983,7710, 7711, 77121, 76071.

^c^ Children too young for testing at time of the available study data.

eFigure S2. Directed Acyclic Graph of possible causal pathways relevant to prenatal antipsychotic exposure and risk of child neurodevelopmental disorders.

The diagram displays measured and adjusted factors (white) with closed (adjusted) confounding pathways (black lines), unmeasured and not adjusted for intermediate variables (blue). Causal pathways denoted by green lines.


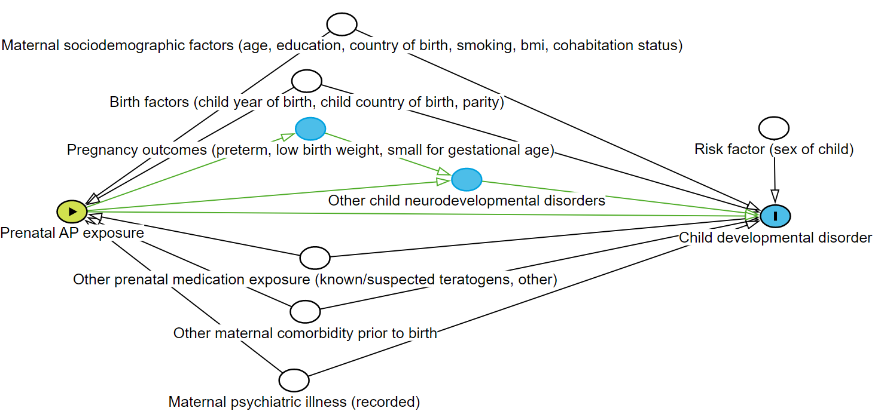

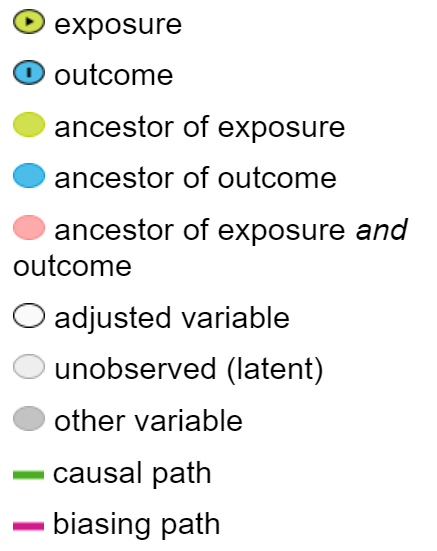

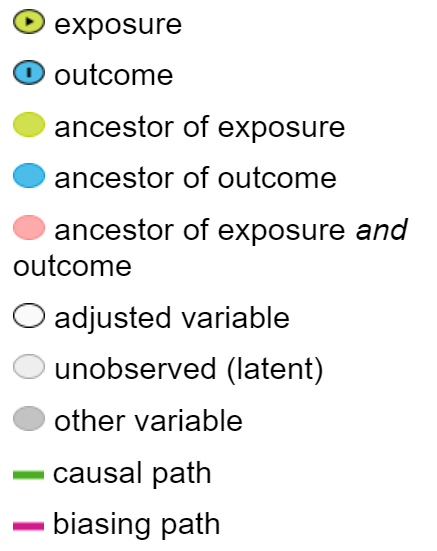

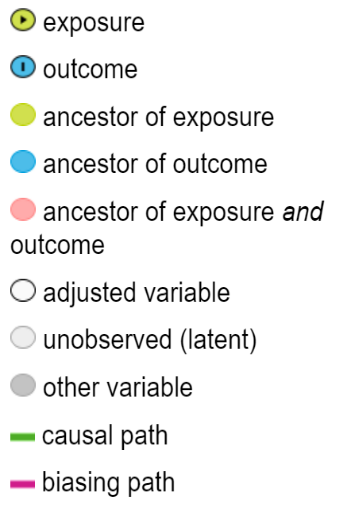

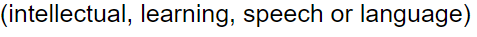

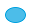

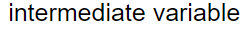


AP, antipsychotic exposure; bmi, body mass index

eFigure S3. Directed Acyclic Graph of possible causal pathways relevant to prenatal antipsychotic exposure and risk of poor school performance A) with potential selection bias and B) addressing selection bias with inverse probability censor weights.

The diagram displays measured and adjusted factors (white) with closed (adjusted) confounding pathways (black lines), unmeasured and not adjusted for intermediate variables (blue). Causal pathways denoted by green lines and bias pathways by pink lines.


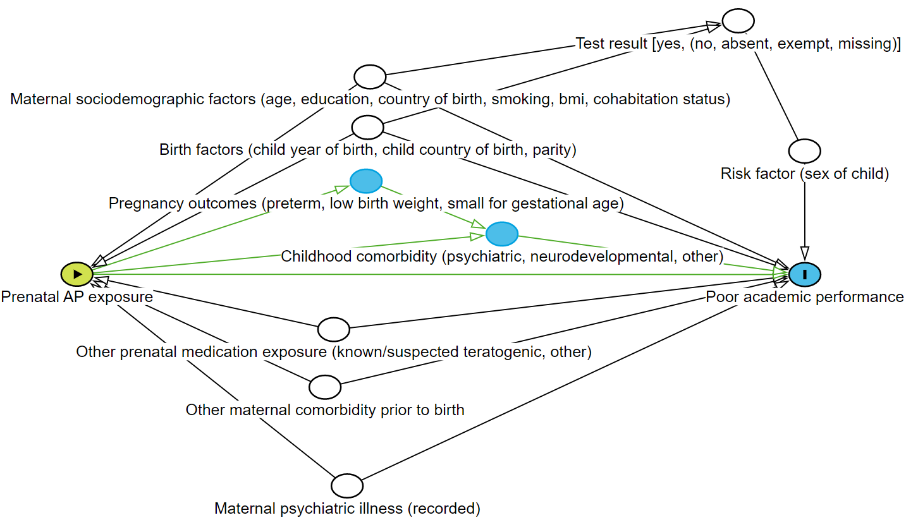

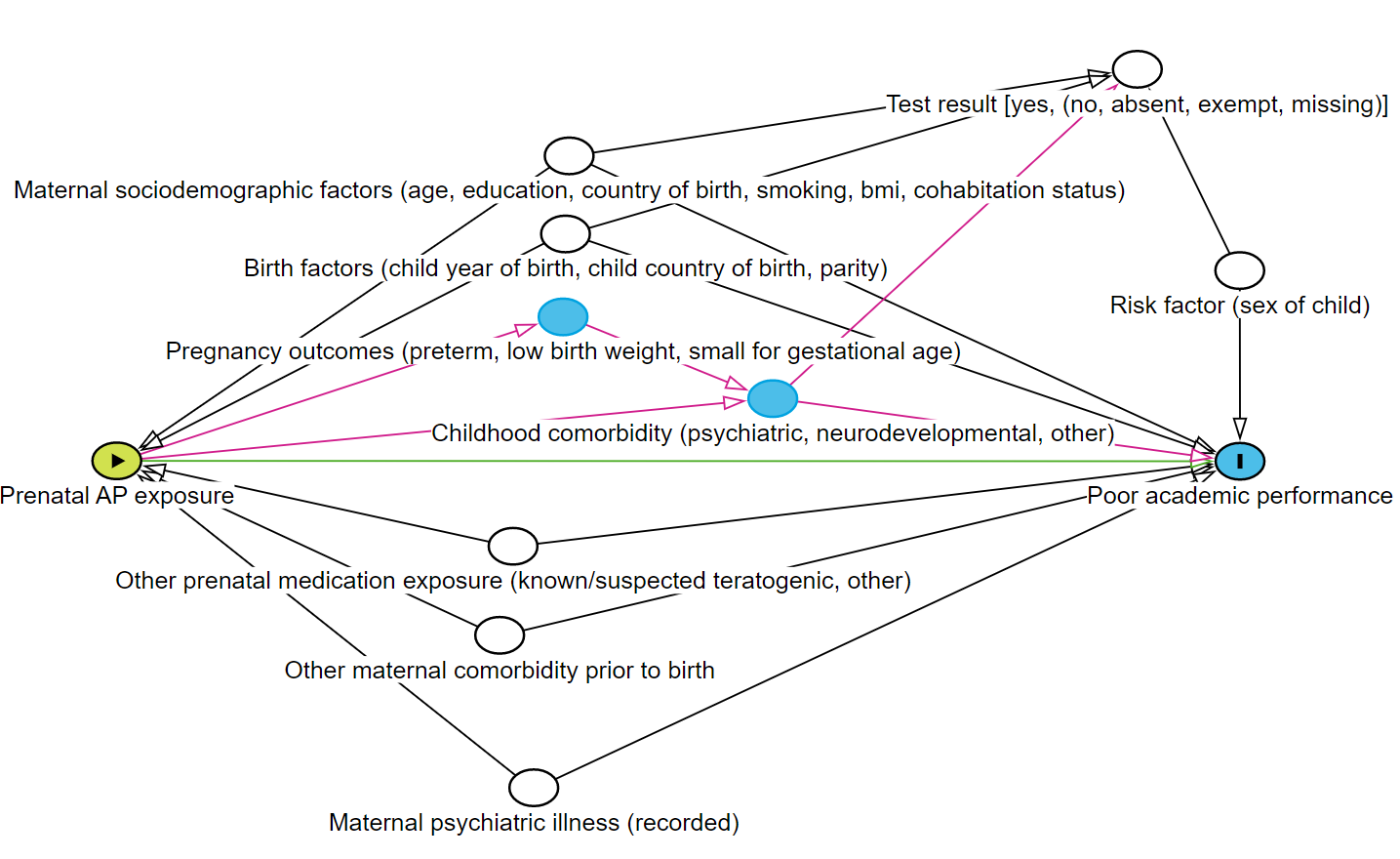


A

B


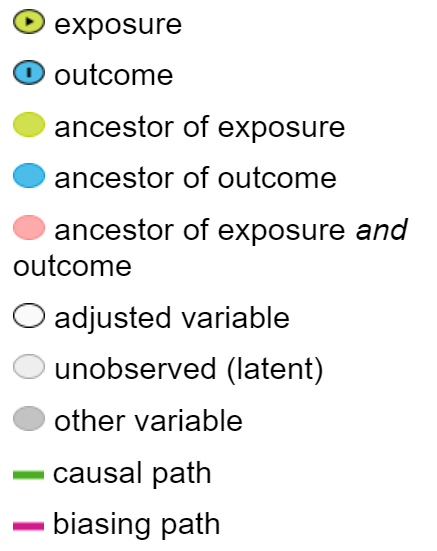

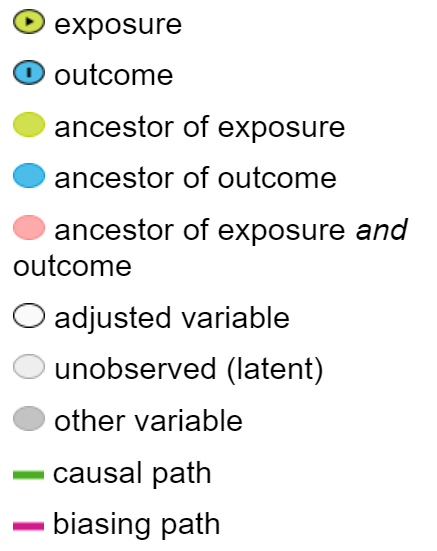

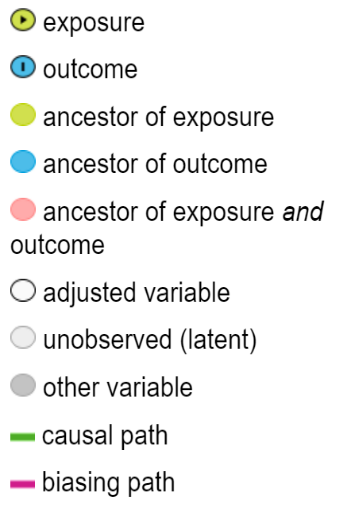

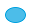

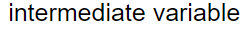

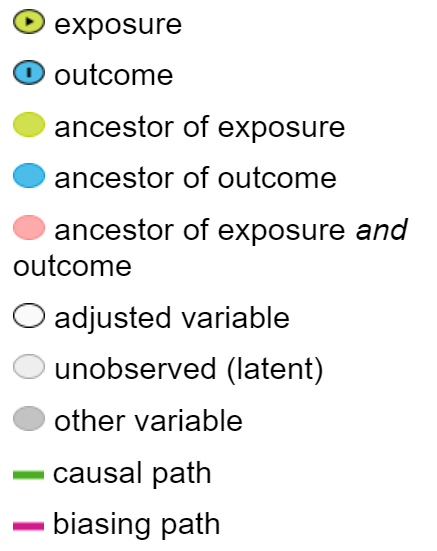

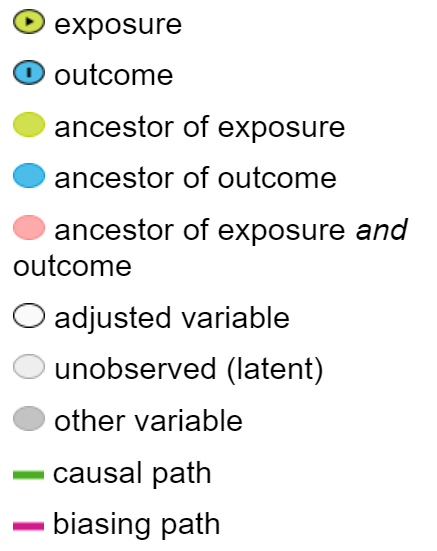

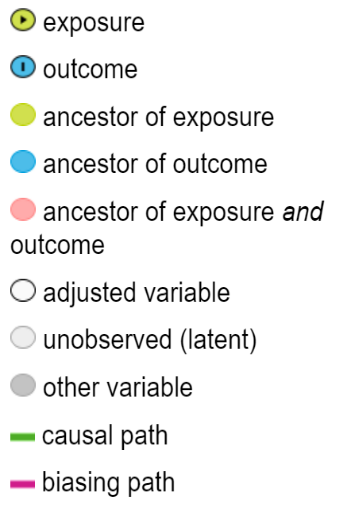

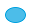

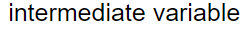


AP, antipsychotic exposure; bmi, body mass index

eTable S1. Ethical boards providing approval for data use & approval numbers.

| Country | Ethical Board and/or data protection authorities | Approval Number |
| --- | --- | --- |
| Denmark | No Ethical Board approval needed. The study is reported to the Danish Data Protection Agency through registration at Aarhus University | KEA‐2016051-000001/ 1833 |
| Finland | No Ethical Board approval needed.  Finnish Institute for Health and Welfare and the Social Insurance Institution of Finland | THL/1551/6.02.00/2018, THL/1673/5.05.00/2019, Kela 117/522/2019 |
| Iceland | National Bioethics Committee | VSNb2018060017/03.01 |
| Norway | Regional Committee for Medical Research Ethics South/East Norway  The Norwegian Data Inspectorate in Norway | 2017/2546/REC South-East  17/02068/Norwegian Data Inspectorate |
| Sweden | Swedish Ethical Review Authority (Etikprövningsmyndigheten) | dnr 2015/1826-31/2, 2017/2238-32, 2018/1790-32, 2018/2211-32 |

The relevant ethical and/or data protection authorities in all countries approved the project and granted a waiver of

informed consent.

eTable S2. Anatomical Therapeutic Chemical classification codes for antipsychotic medications included in study.

| ATC | Exposure |
| --- | --- |
| N05A | Antipsychotics |
| N05AA | Phenothiazines with aliphatic side-chain |
| N05AA01 | chlorpromazine |
| N05AA02 | levomepromazine |
| N05AA04 | acepromazine |
| N05AB | Phenothiazines with piperazine structure |
| N05AB01 | dixyrazine |
| N05AB02 | fluphenazine |
| N05AB03 | perphenazine |
| N05AB04 | prochlorperazine |
| N05AC | Phenothiazines with piperidine structure |
| N05AC01 | periciazine |
| N05AC02 | thioridazine |
| N05AD | Butyrophenone derivatives |
| N05AD01 | haloperidol |
| N05AD03 | melperone |
| N05AD05 | pipamperone |
| N05AE | Indole derivatives |
| N05AE03 | sertindole |
| N05AE04 | ziprasidone |
| N05AE05 | lurasidone |
| N05AF | Thioxanthene derivatives |
| N05AF01 | flupentixol |
| N05AF03 | chlorprothixene |
| N05AF05 | zuclopenthixol |
| N05AG | Diphenylbutylpiperidine derivatives |
| N05AG02 | pimozide |
| N05AG03 | penfluridol |
| N05AH | Diazepines, oxazepines, thiazepines and oxepines |
| N05AH02 | clozapine |
| N05AH03 | olanzapine |
| N05AH04 | quetiapine |
| N05AH05 | asenapine |
| N05AL | Benzamides |
| N05AL01 | sulpiride |
| N05AL03 | tiapride |
| N05AL05 | amisulpride |
| N05AX | Other antipsychotics |
| N05AX08 | risperidone |
| N05AX12 | aripiprazole |
| N05AX13 | paliperidone |
| N05AX15 | cariprazine |
| N05AX16 | brexpiprazole |

ATC, Anatomical Therapeutic Chemical

eTable S3. Exposure and covariate definitions, source data, and relevant diagnostic, medication codes.

| **Covariate/Exposure** | **Time frame** | **Functional form of variable and comments** | **Data source ^a^** | **Codes** | | |
| --- | --- | --- | --- | --- | --- | --- |
|  |  |  |  | **ATC** | **ICD-10** | **ICPC-2** |
| Anytime during pregnancy exposure | LMP to birth minus 1 day | Categorical: Y/N. ≥1 prescription fills for any of listed medications | MBR PDR | N05B |  |  |
| Late pregnancy exposure | LMP+98 days to birth minus 1 day | Categorical: Y/N. ≥1 prescription fills for any of listed medications | MBR PDR | N05B |  |  |
| Throughout pregnancy exposure | LMP to 97 days & LMP+98 days to birth minus 1 day | Categorical: Y/N. ≥1 prescription fills for any of listed medications during both time windows | MBR PDR | N05B |  |  |
| Calendar year of delivery | Year of birth | Continuous | MBR |  |  |  |
| Maternal age | At delivery | Categorical: <20; 20-24; 25-29; 30-34; 35-39; ≥40 | MBR |  |  |  |
| Maternal education | Year of delivery | Ordinal: compulsory education or less (ISCED: 0,1); secondary education (ISCED: 3,4); post-secondary education (ISCED: 5,6,7,8); missing. Finland: not available | NSI |  |  |  |
| Cohabitation status^b^ | Year of delivery | Cohabiting.  Categorical: Y/N/missing | MBR |  |  |  |
| Maternal country of birth | n/a | Mother born in the country of delivery or not. Categorical: Y/N/missing | NSI MBR |  |  |  |
| Parity | n/a | Number of previous deliveries.  Categorical: 0, 1, ≥2 or missing | MBR |  |  |  |
| Maternal BMI ^a^ | In early pregnancy | Continuous. Categorical. <18.5, 18.5 - <25, 25 - <30, ≥ 30; missing. Set to missing for those with BMI: <14 or > 55. Missing/invalid, recorded as missing. | MBR |  |  |  |
| Smoking | In early pregnancy | Categorical: Y/N/Missing Iceland: not available | MBR |  |  |  |
| Child sex | At delivery | Categorical: Female; Male | MBR |  |  |  |
| **Maternal comorbidity** | **LMP-365 to delivery** | **Categorical: Y/N.**  **≥1 diagnosis of listed conditions** | **NPR MBR** |  |  |  |
| Pre-existing diabetes |  |  |  |  | E10-E14 | T89, T90, W85 |
| Pre-existing hypertension |  |  |  |  | I10-I15, O10 | K86, K87 |
| Polycystic ovary syndrome |  |  |  |  | E28.2 |  |
| Migraine |  |  |  |  | G43 | N89 |
| Cluster headache |  |  |  |  | G44 | N90 |
| Epilepsy or convulsion, or epilepsy complicating pregnancy |  |  |  |  | G40,  G41 | N88 |
| **Maternal psychiatric conditions** | **LMP-365 to delivery** | **Categorical: Main, other, reimbursed. ≥1 diagnosis of listed conditions or  ≥1 prescription fills for any of listed medications with reimbursement code** | **NPR MBR PDR** |  |  |  |
| Bipolar disorders |  | Main |  |  | F30-F31 |  |
| Schizophrenia and other psychotic disorders |  | Main |  |  | F20-F29 |  |
| Substance use disorder |  | Other |  |  | F10-F19 |  |
| Depression and other mood disorders, excl. bipolar disorder |  | Other |  |  | F32-F39 |  |
| Anxiety disorders |  | Other |  |  | F40-F48 |  |
| Personality disorders |  | Other |  |  | F60-F69 |  |
| Intellectual developmental disorder |  | Other |  |  | F70-F79 |  |
| Disorders of psychological development |  | Other |  |  | F80-F89 |  |
| Autism spectrum disorder |  | Other |  |  | F84.0, F84.1, F84.5 |  |
| Attention-deficit/hyperactivity disorder |  | Other |  |  | F90.0-F90.2, F90.8, F90.9 |  |
| Suicide attempts |  | Other |  |  | X60-X84, Y10-Y34 |  |
| Psychiatric disorder as recorded as the indication for prescription reimbursement ^c^ |  | Reimbursed |  | N0 w/ reimbursement code 18 |  |  |
| **Known/suspected teratogens use** | **LMP-90 to delivery** | **Categorical: Y/N. ≥1 prescription fills for any of listed medications** | **PDR** |  |  |  |
| Warfarin |  |  |  | B01AA03 |  |  |
| Antineoplastic agents |  |  |  | L01 |  |  |
| Isotretinoin |  |  |  | D10AD04 D10BA01 D10AD54 |  |  |
| Systemic retinoids for psoriasis dermatitis |  |  |  | D05BB D11AH04 |  |  |
| Misoprostol |  |  |  | A02BB01 G02AD06 M01AB55 M01AE56 |  |  |
| Thalidomide |  |  |  | L04AX02 |  |  |
| Valproate |  |  |  | N03AG01 |  |  |
| Lithium |  |  |  | N05AN01 |  |  |
| Antiepileptics [excl. valproate] |  |  |  | N03 [excl. N03AG01] |  |  |
| Drugs acting on the renin-angiotensin system |  |  |  | C09 |  |  |
| Antithyroid drugs |  |  |  | H03B |  |  |
| Mycophenolic acid |  |  |  | L04AA06 |  |  |
| Leflunomide |  |  |  | L04AA13 |  |  |
| Teriflunomide |  |  |  | L04AA31 |  |  |
| Lenalidomide |  |  |  | L04AX04 |  |  |
| Pomalidomide |  |  |  | L04AX06 |  |  |
| Ergot alkaloids |  |  |  | N02C |  |  |
| **Other medication use** | **LMP-90 to delivery** | **Categorical: Y/N.**  **≥1 prescription fills for any of listed medications** | **PDR** |  |  |  |
| Antidepressants |  |  |  | N06A |  |  |
| Antidiabetics |  |  |  | A10 |  |  |
| Benzodiazepines |  |  |  | N05BA N05CD |  |  |
| Triptans |  |  |  | N02CC |  |  |
| Opioids |  |  |  | N02A |  |  |
| Paracetamol |  |  |  | N02BE01 |  |  |

ATC, anatomical therapeutic chemical classification system; DK, Denmark; FI, Finland; IS, Iceland; ICD-10, International statistical classification of diseases and related health conditions, revision 10; ISCED, International classification for education; LMP, last menstrual period; MBR, Medical birth register; NPR, National patient register; NSI, National statistical institutes; NO, Norway; PDR, Prescribed drug register; SE, Sweden

^a^ Recorded in MBR or calculated from maternal weight and height at first antenatal visit (kg/m^2^)

^b^ In Finland, Iceland, Norway and Sweden, cohabitating refers to any situation where the mother reports living with a partner, in Denmark cohabiting refers to married or registered partnerships only.

^c^ Psychiatric disorder recorded as the indication for prescription using reimbursement codes defined by the Norwegian Medicines Agency for chronic psychiatric disorders for women in Norway with births between 2005 and 2010.

eTable S4. Multiple imputations by chained equations.

| **Outcome (s)** | Model: imputed variables | Predictors included in model | Number of imputations |
| --- | --- | --- | --- |
| All neurodevelopmental outcomes | Binary logistic regression: Smoking, maternal birth country, cohabitation status  Ordinal logistic regression: maternal education, parity  Predictive mean modelling: BMI | All covariates ^a^  Antipsychotic exposure during pregnancy (binary: yes/no)  Composite neurodevelopmental outcome (binary: yes/no)  Follow-up time (days) | 20 |
| Poor academic performance in mathematics | Binary logistic regression: Smoking, maternal birth country, cohabitation status  Ordinal logistic regression: maternal education, parity  Predictive mean modelling: BMI | All covariates ^a^  Antipsychotic exposure during pregnancy (binary: yes/no)  Poor academic performance in mathematics (binary: yes/no) | 40 |
| Poor academic performance in language arts | Binary logistic regression: Smoking, maternal birth country, cohabitation status  Ordinal logistic regression: maternal education, parity  Predictive mean modelling: BMI | All covariates ^a^  Antipsychotic exposure during pregnancy (binary: yes/no)  Poor academic performance in language arts (binary: yes/no) | 40 |

Missing values for body mass index (BMI), maternal education, smoking status in early pregnancy, maternal birth country, marital/cohabitation status, and parity were imputed using multiple imputation by chained equations (MICE). ^5^ All covariates used in regression models were included as predictors, as well as the listed exposure and outcome variables. Analyses were conducted on these imputed datasets using Rubin’s rule. ^6^ Multiple imputation was conducted in R using the *mice* package ^7^ and a seed value of *9999*.

BMI, body mass index

^a^ All covariates listed in eTable 3.

eTable S5. Crude cumulative incidence (with 95% confidence intervals) for each neurodevelopmental disorder in children of mothers with psychiatric disorders.

| Neurodevelopmental disorder | Cumulative risk (%) at age 6 (95% CI) | Cumulative risk (%) at age 8  (95% CI) | Mean (SD) age at diagnosis | Mean (SD) duration of follow-up |
| --- | --- | --- | --- | --- |
| Finnish, Icelandic, Norwegian, and Swedish cohort |  |  |  |  |
| Developmental intellectual disorder | 0.2 (0.2 – 0.2) | 0.5 (0.4 – 0.5) | 7.6 (3.0) | 7.1 (4.5) |
| Learning disorder ^a^ | 0.1 (0.1 – 0.1) | 0.4 (0.3 – 0.4) | 10.0 (2.7) | 7.2 (4.6) |
| Speech/language disorder | 1.2 (1.1 – 1.2) | 1.6 (1.5 – 1.7) | 5.5 (2.1) | 7.2 (4.5) |
| Composite neurodevelopmental disorder | 1.5 (1.4 – 1.5) | 2.3 (2.2 – 2.4) | 7.2 (3.2) | 7.2 (4.5) |
| Danish cohort |  |  |  |  |
| Developmental intellectual disorder | 0.3 (0.3 – 0.4) | 0.6 (0.5 – 0.7) | 7.9 (4.1) | 8.1 (4.8) |
| Learning disorder ^a^ | NA | NA | 11.4 (3.0) | 8.2 (4.9) |
| Speech/language disorder | 0.2 (0.2 – 0.3) | 0.4 (0.3 – 0.5) | 6.4 (2.7) | 8.2 (4.9) |
| Composite neurodevelopmental disorder | 0.6 (0.5 – 0.7) | 1.0 (0.8 – 1.1) | 8.1 (3.9) | 8.1 (4.8) |

NA, Not Applicable (too few numbers); CI, confidence interval; SD, standard deviation

Cumulative incidences from combined cohort (Finland, Iceland, Norway, Sweden) and Danish cohort.

^a^ Follow up time starts from age of 5 years, all others begin age of 3 years.

eTable S6. Secondary analysis: hazard ratios and risk ratios (with 95% confidence intervals) for child neurodevelopmental disorders and poor academic performance when comparing children prenatal exposed with children whose mothers discontinued prior to pregnancy.

|  | Exposed in pre-pregnancy only | Exposed any time during pregnancy | Hazard Ratio/Risk Ratio (95% CI) | | |
| --- | --- | --- | --- | --- | --- |
|  | No of events/No of pregnancies | No of events/No of pregnancies | Minimally adjusted ^a^ | Fully adjusted ^b^ | Fully adjusted ^b^ |
| Developmental intellectual disorder | 33/6713 | 51/8155 | 1.18 (0.61 – 2.29) | 1.05 (0.57 – 1.94) | 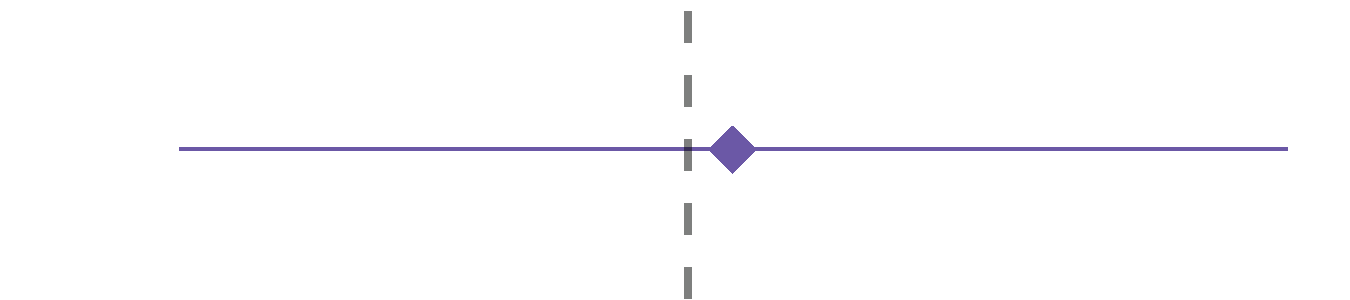 |
| Learning disorder ^c^ | 29/4908 | 39/6205 | 1.13 (0.70 – 1.83) | 1.03 (0.63 – 1.69) | 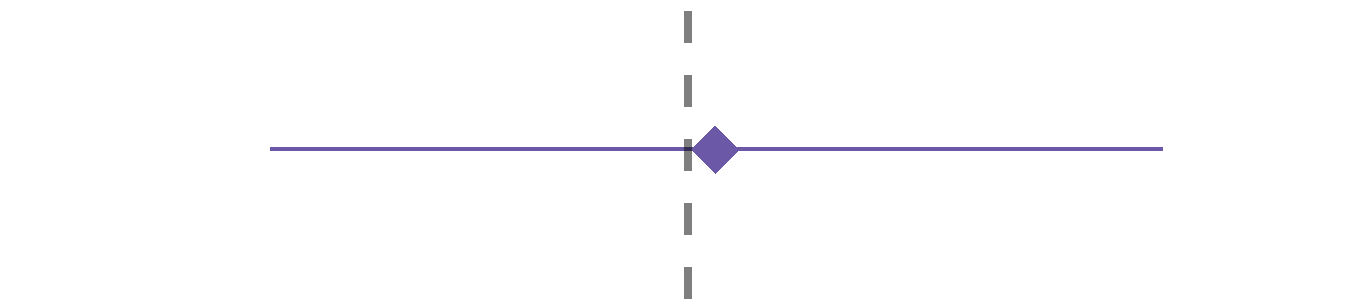 |
| Speech or language disorder ^c^ | 33/4908 | 43/6205 | 1.15 (0.72 – 1.84) | 1.02 (0.64 – 1.63) | 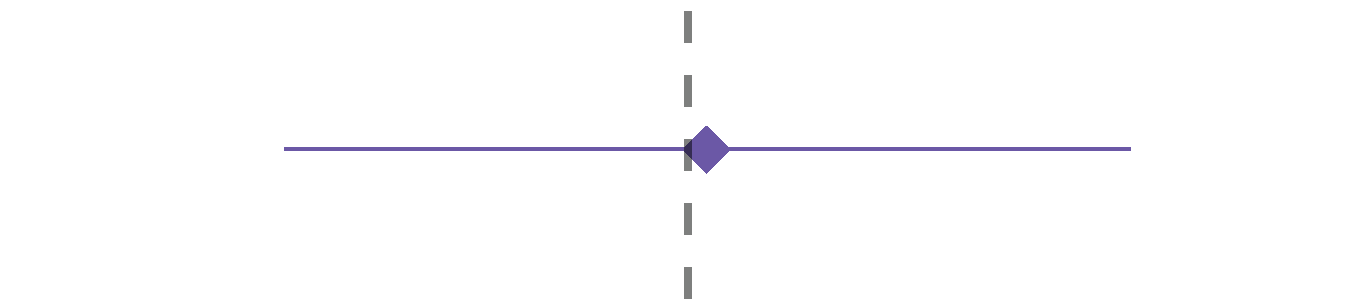 |
| Composite neurodevelopmental outcome | 103/6713 | 134/8155 | 0.95 (0.57 – 1.60) | 0.93 (0.61 – 1.40) | 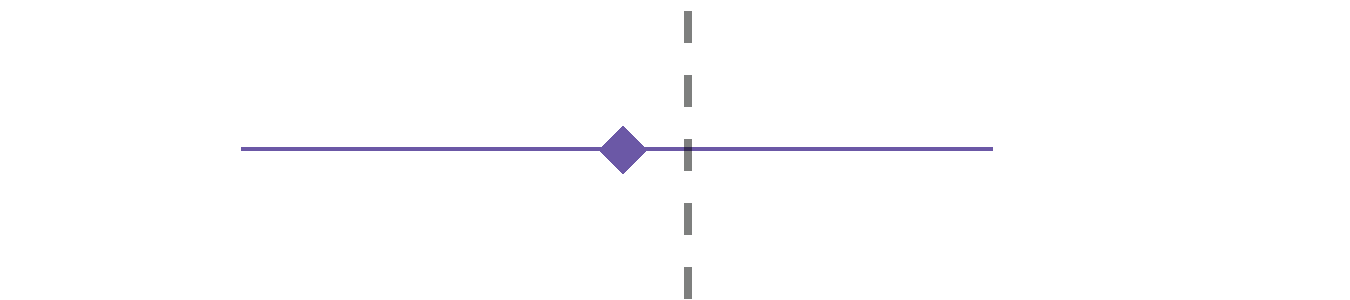 |
| Poor performance in mathematics | 571/1521 | 751/1906 | 1.03 (0.94 – 1.13) | 1.02 (0.93 – 1.12) | 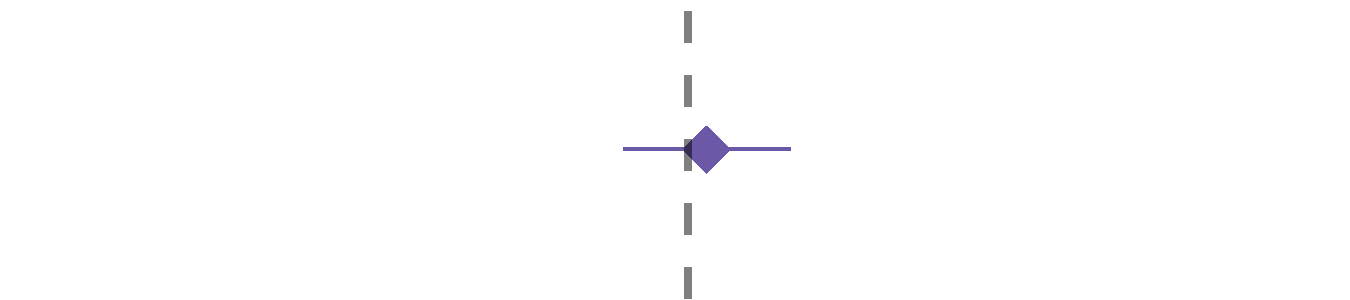 |
| Poor performance in language arts | 502/1478 | 625/1879 | 0.97 (0.89 – 1.07) | 0.95 (0.86 – 1.05) | 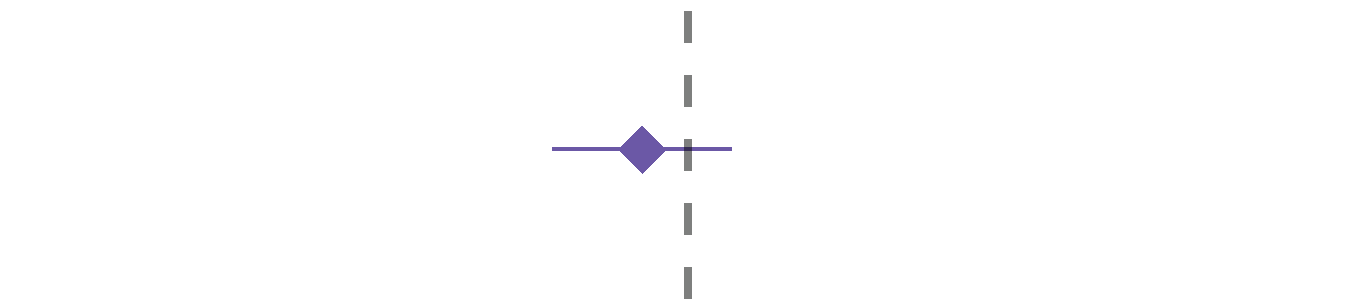 |
|  |  |  |  |  | 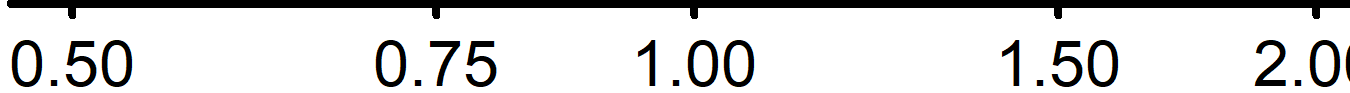 |

BMI, body mass index; CI, confidence interval

Finland was not included in this analysis due to data availability.

^a^ Adjusted for birth year and sex of child, child’s country of birth, maternal age, parity using outcome regression.

^b^ Adjusted for birth year, sex of child, child’s country of birth of child, maternal country of birth, age, parity, education, cohabitation status, BMI & smoking in early pregnancy, use of other medications during pregnancy, or known/suspected teratogens and comorbidity prior to pregnancy using propensity score overlap weights.

^c^ Results presented only for the combined cohort (Iceland, Norway and Sweden), owing to low number of exposure/outcomes in the Danish cohort.

eTable S7. Sensitivity analysis: hazard ratios and risk ratios (with 95% confidence intervals) for child neurodevelopmental disorder and poor academic performance when redefining exposure as at least two prescription fills any time during pregnancy.

|  | **Unexposed** | **≥2 prescription fills during pregnancy** | **Hazard Ratio/Risk Ratio (95% CI)** | | |
| --- | --- | --- | --- | --- | --- |
|  | **No of events/No of pregnancies** | **No of events/No of pregnancies** | **Minimally adjusted ^a^** | **Fully adjusted ^b^** | **Fully adjusted ^b^** |
| Developmental intellectual disorder | 869/197 296 | 48/6984 | 1.55 (1.15 – 2.11) | 1.10 (0.78 – 1.55) | 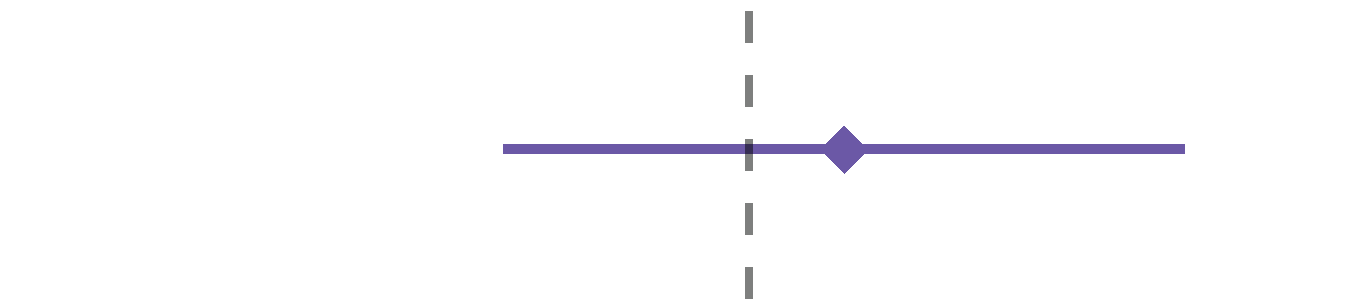 |
| Learning disorder ^c^ | 1304/163 604 | 79/5805 | 1.42 (1.13 – 1.79) | 1.09 (0.84 – 1.41) | 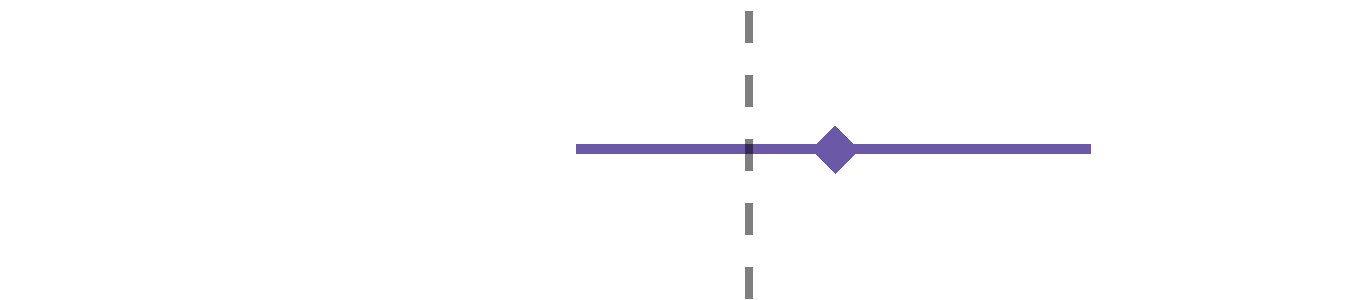 |
| Speech or language disorder ^c^ | 1773/163 604 | 125/5805 | 1.27 (1.05 – 1.53) | 1.00 (0.81 – 1.24) | 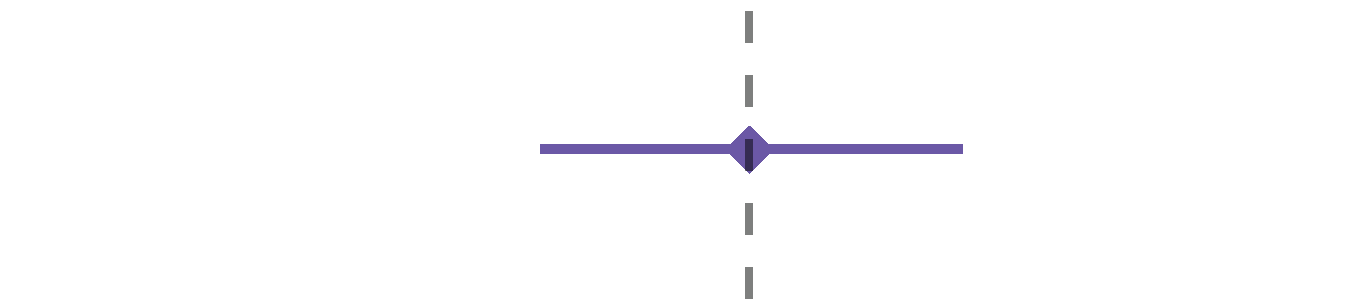 |
| Composite neurodevelopmental outcome | 3823/197 296 | 239/6984 | 1.36 (1.19 – 1.56) | 1.06 (0.91 – 1.24) | 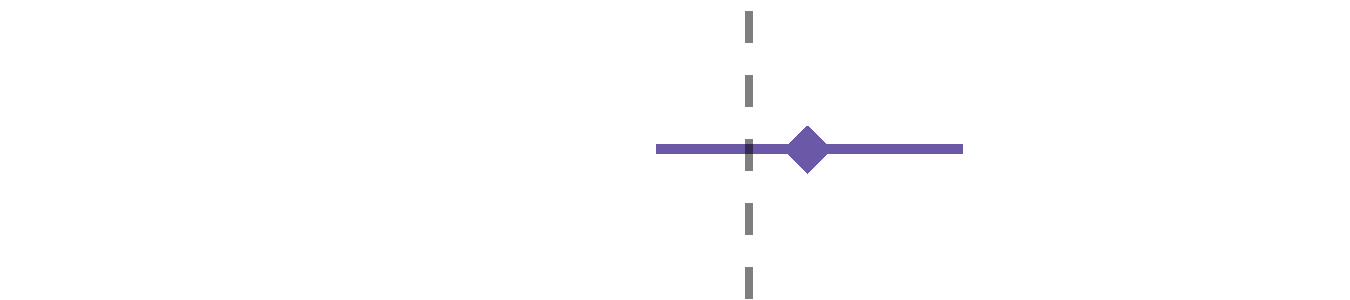 |
| Poor performance in mathematics ^d^ | 13 190/38 088 | 414/1011 | 1.21 (1.12 – 1.31) | 1.07 (0.98 – 1.17) | 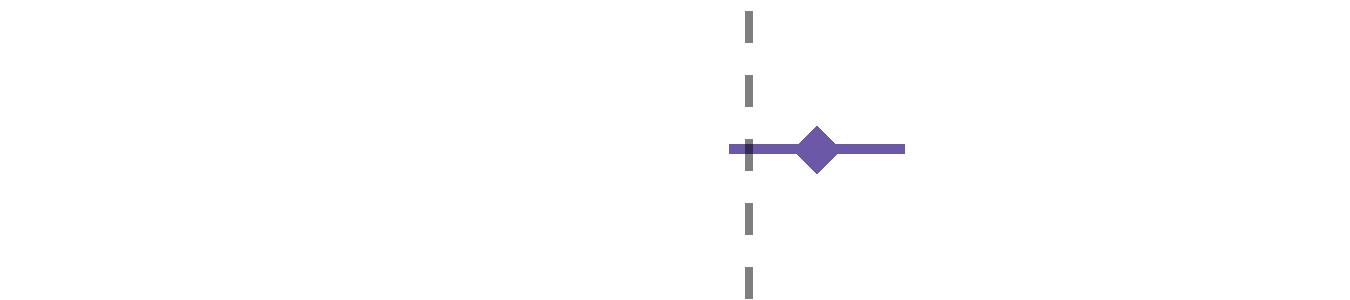 |
| Poor performance in language arts ^d^ | 11 105/37 541 | 341/997 | 1.17 (1.07 – 1.28) | 1.03 (0.93 – 1.14) | 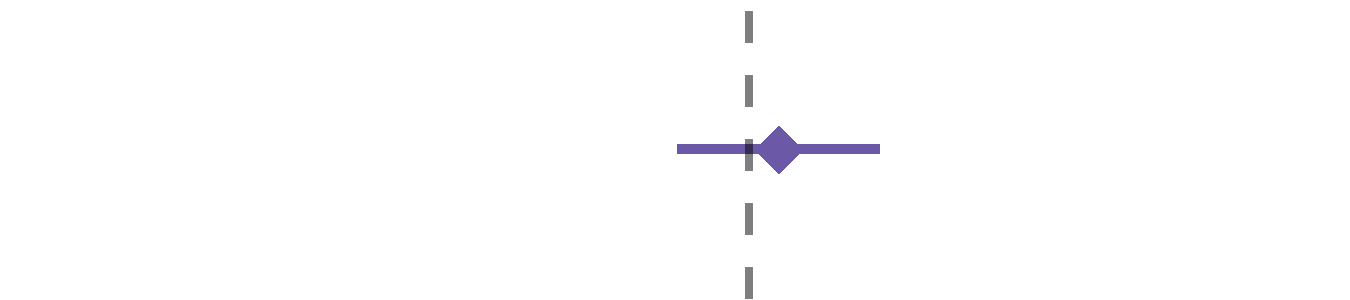 |
|  |  |  |  |  | 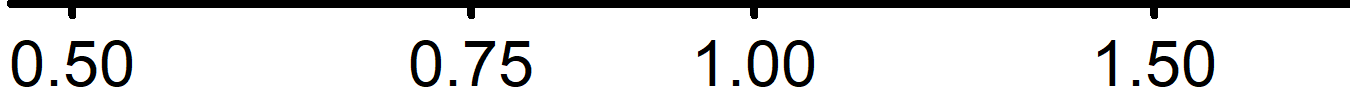 |

BMI, body mass index; CI, confidence interval

^a^ Adjusted for birth year and sex of child, child’s country of birth, maternal age, parity using outcome regression.

^b^ Adjusted for birth year, sex of child, child’s country of birth of child, maternal country of birth, age, parity, education, cohabitation status, BMI & smoking in early pregnancy, use of other medications during pregnancy, or known/suspected teratogens and comorbidity prior to pregnancy using propensity score overlap weights.

^c^ Results presented only for the combined cohort (Finland, Iceland, Norway and Sweden), owing to low number of exposure/outcomes in the Danish cohort.

^d^ Finland was not included in this analysis due to data availability.

eTable S8. Sensitivity analysis: risk ratios (with 95% confidence intervals) for poor academic performance after prenatal antipsychotic exposure by timing of exposure when redefining poor performance outcome as scoring in the lowest 10th percentile.

|  |  | **Risk Ratio (95% CI)** | | |
| --- | --- | --- | --- | --- |
|  | **No of events /No of pregnancies** | **Minimally adjusted ^a^** | **Fully adjusted ^b^** | **Fully adjusted ^b^** |
| **Mathematics** |  |  |  |  |
| Unexposed | 3422/25 740 | Reference | Reference | 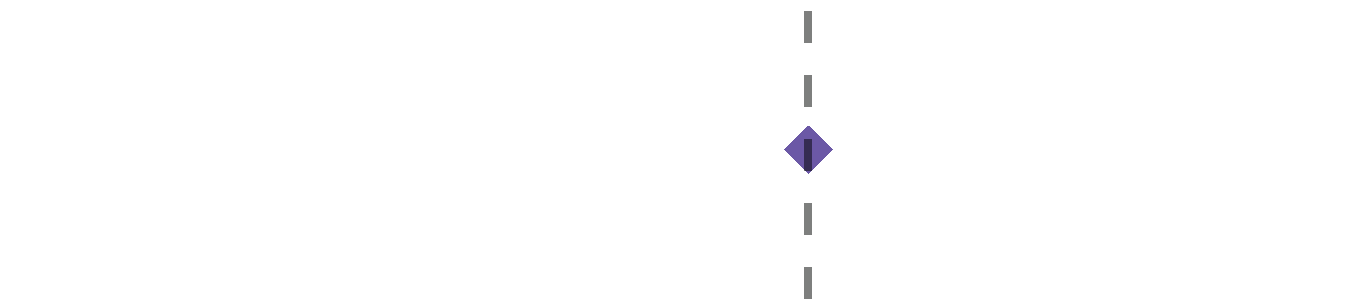 |
| Exposed any time | 226/1436 | 1.15 (1.05 – 1.27) | 1.02 (0.89 – 1.16) | 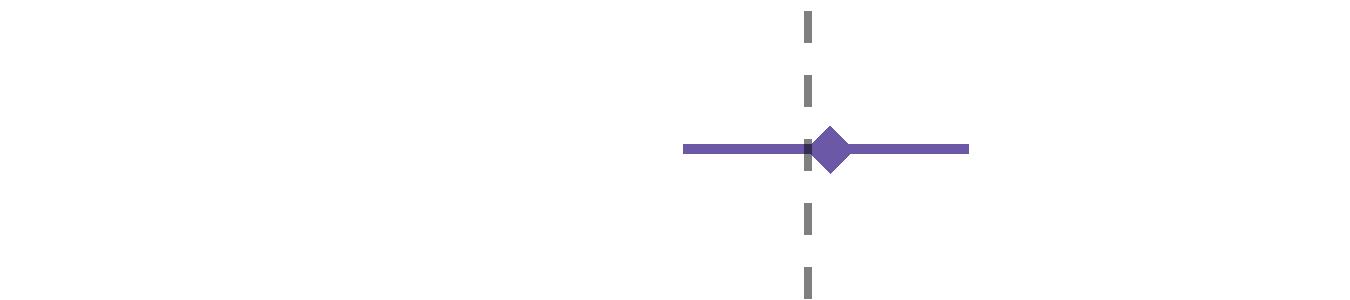 |
| Exposed in late pregnancy only | 45/298 | 1.05 (0.84 – 1.31) | 0.98 (0.73 – 1.33) | 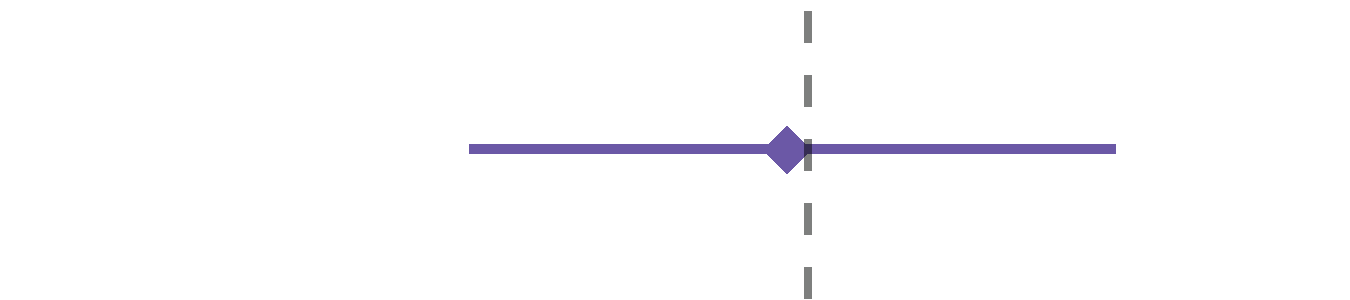 |
| Exposed throughout pregnancy | 93/492 | 1.36 (1.18 – 1.57) | 1.21 (0.97 – 1.50) | 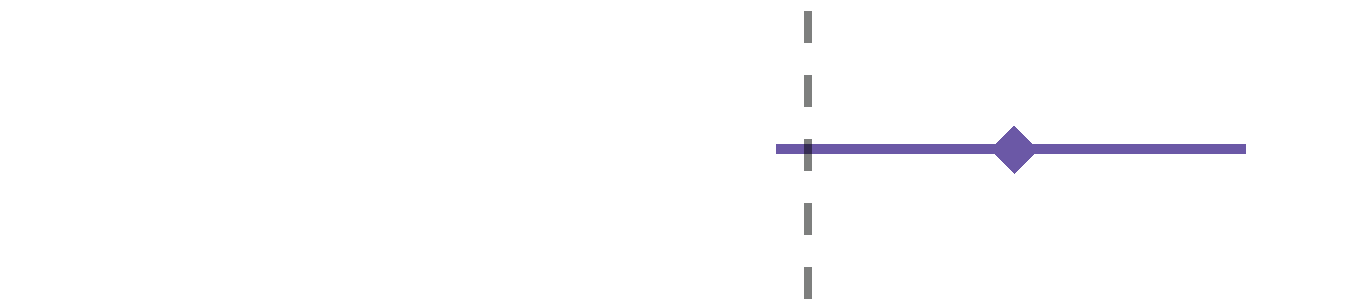 |
| **Language Arts** | | | | |
| Unexposed | 2937/25 190 | Reference | Reference | 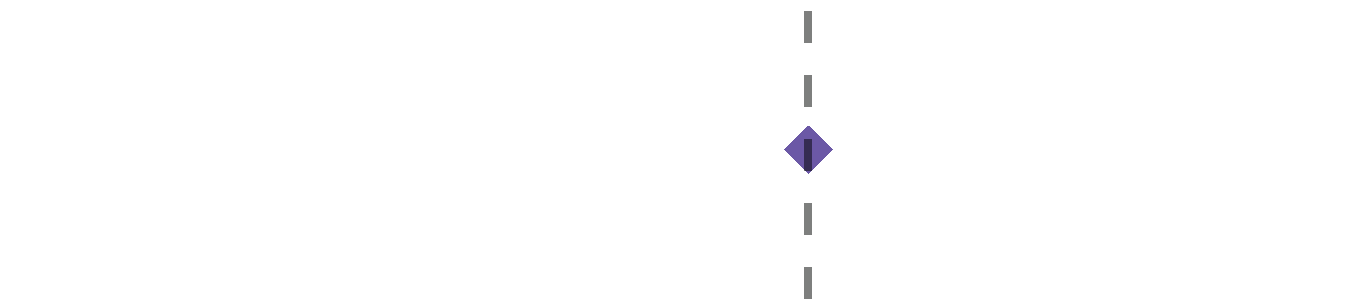 |
| Exposed any time | 212/1410 | 1.17 (1.05 – 1.29) | 1.04 (0.90 – 1.20) | 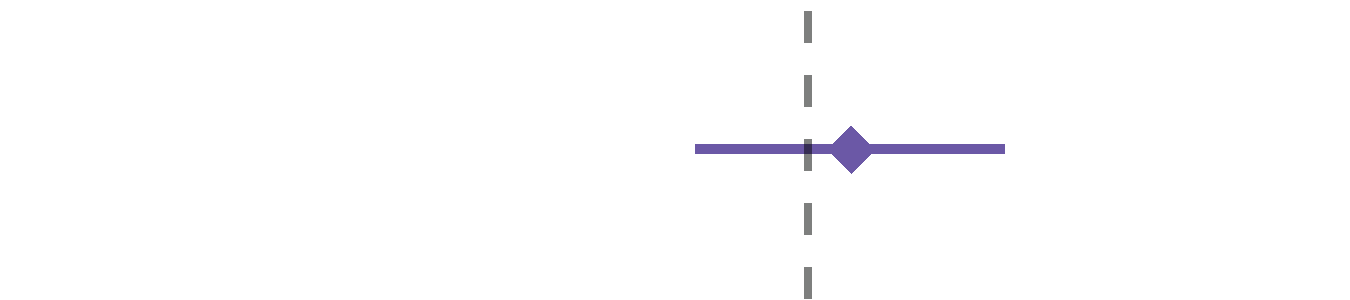 |
| Exposed in late pregnancy only | 41/285 | 1.21 (0.96 – 1.52) | 1.12 (0.81 – 1.56) | 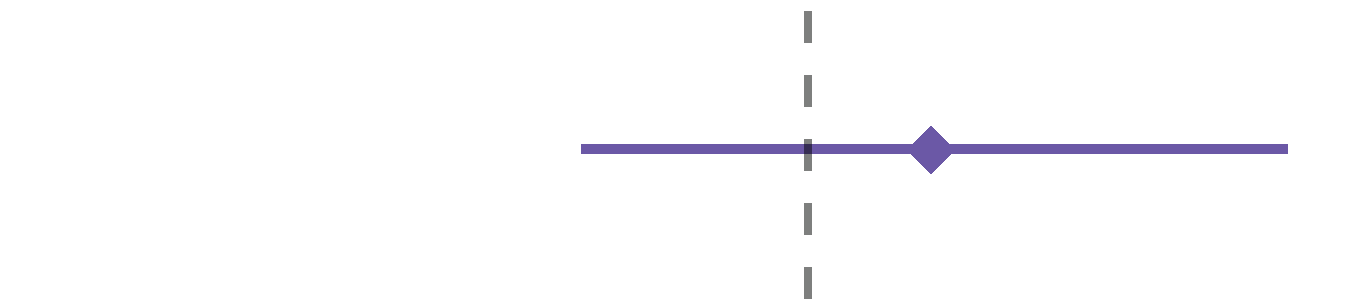 |
| Exposed throughout pregnancy | 81/487 | 1.22 (1.03 – 1.44) | 1.09 (0.85 – 1.39) | 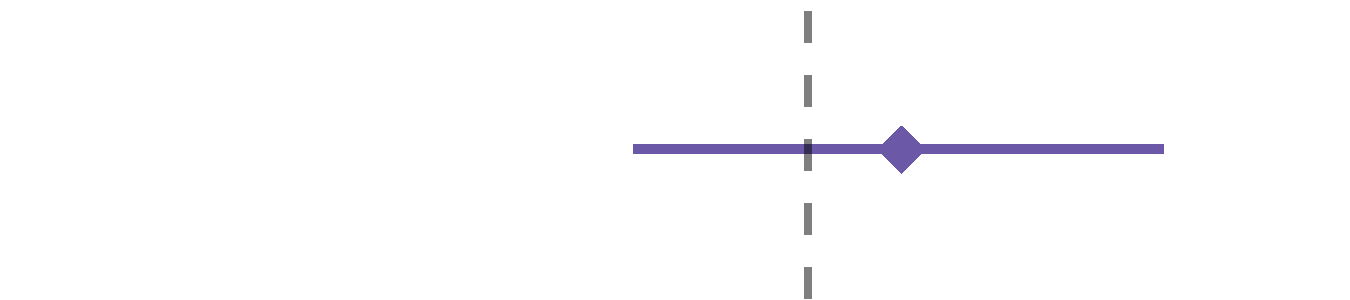 |
|  |  |  |  | 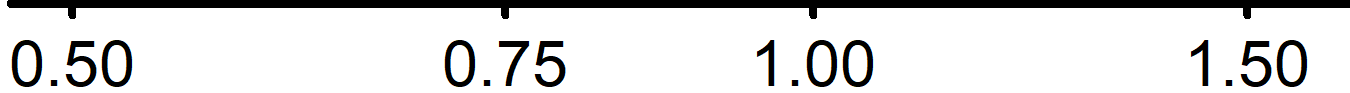 |

BMI, body mass index; CI, confidence interval

Finland was not included in this analysis due to data availability.

^a^ Adjusted for birth year and sex of child, child´s country of birth, maternal age, parity using outcome regression.

^b^ Adjusted for birth year, sex of child, child´s country of birth, maternal birth country, age, parity, education, cohabitation status, BMI & smoking in early pregnancy, use of other medications during pregnancy, or known/suspected teratogens and comorbidity prior to pregnancy using propensity score overlap weights.

eTable S9. Comparison of maternal and child characteristics for children with and without a national standardised test result born between 2000 and 2011.

|  | **Icelandic, Norwegian, and Swedish cohort** | | | **Danish cohort** | | |
| --- | --- | --- | --- | --- | --- | --- |
|  | **Test result** (n=30 500) | **No test result** (n=2522) | **SMD** | **Test result** (n=10 469) | **No test result** (n=2261) | **SMD** |
| **Maternal characteristics** | **n (%)** | **n (%)** |  | **n (%)** | **n (%)** |  |
| **Exposure during pregnancy** |  |  |  |  |  |  |
| Unexposed | 29 120 (95.5%) | 2370 (94.0%) | 0.067 | 9891 (94.5%) | 2141 (94.7%) | 0.009 |
| Exposed any time | 1380 (4.5%) | 152 (6.0%) |  | 578 (5.5%) | 120 (5.3%) |  |
| **Maternal psychiatric diagnosis** | | |  |  |  |  |
| Psychotic or bipolar disorders | 1336 (4.4%) | 110 (4.4%) | 0.052 | 679 (6.5%) | 174 (7.7%) | 0.047 |
| Other | 21 211 (69.5%) | 1696 (67.2%) |  | 9790 (93.5%) | 2087 (92.3%) |  |
| Psychiatric disorders recorded by prescription reimbursement ^a^ | 7953 (26.1%) | 716 (28.4%) |  | - | - |  |
| **Age at delivery, years** |  |  |  |  |  |  |
| <20 | 1246 (4.1%) | 127 (5.0%) | 0.087 | 504 (4.8%) | 75 (3.3%) | 0.181 |
| 20–24 | 5552 (18.2%) | 513 (20.3%) |  | 2208 (21.1%) | 362 (16.0%) |  |
| 25–29 | 8246 (27.0%) | 672 (26.6%) |  | 3138 (30.0%) | 683 (30.2%) |  |
| 30–34 | 8992 (29.5%) | 676 (26.8%) |  | 2895 (27.7%) | 660 (29.2%) |  |
| 35–39 | 5219 (17.1%) | 419 (16.6%) |  | 1451 (13.9%) | 401 (17.7%) |  |
| ≥ 40 | 1245 (4.1%) | 115 (4.6%) |  | 273 (2.6%) | 80 (3.5%) |  |
| **Maternal education in year of delivery** | |  |  |  |  |  |
| Compulsory | 8691 (28.5%) | 1052 (41.7%) | 0.334 | 4508 (43.1%) | 786 (34.8%) | 0.191 |
| Secondary | 12 364 (40.5%) | 854 (33.9%) |  | 4013 (38.3%) | 889 (39.3%) |  |
| Post-secondary | 8939 (29.3%) | 478 (19.0%) |  | 1805 (17.2%) | 525 (23.2%) |  |
| *Missing* | 506 (1.7%) | 138 (5.5%) |  | 143 (1.4%) | 61 (2.7%) |  |
| **Cohabiting** |  |  |  |  |  |  |
| Yes | 24 904 (81.7%) | 1882 (74.6%) | 0.173 | 4538 (43.3%)**^b^** | 1160 (51.3%) | 0.16 |
| No | 5464 (17.9%) | 630 (25.0%) |  | 5931 (56.7%) | 1101 (48.7%) |  |
| *Missing* | 132 (0.4%) | 10 (0.4%) |  | - | - |  |
| **Maternal country of birth** |  |  |  |  |  |  |
| Within country of delivery | 25 799 (84.6%) | 1753 (69.5%) | 0.373 | 9062 (86.6%) | 1785 (78.9%) | 0.203 |
| Outside country of delivery | 4522 (14.8%) | 760 (30.1%) |  | 1407 (13.4%) | 476 (21.1%) |  |
| *Missing* | 179 (0.6%) | 9 (0.4%) |  | - | - |  |
| **BMI, early pregnancy** |  |  |  |  |  |  |
| <18.5 | 606 (2.0%) | 56 (2.2%) | 0.161 | 458 (4.4%) | 83 (3.7%) | 0.087 |
| 18.5–24 | 9,089 (29.8%) | 604 (23.9%) |  | 3,421 (32.7%) | 715 (31.6%) |  |
| 25–29 | 4,117 (13.5%) | 298 (11.8%) |  | 1,474 (14.1%) | 303 (13.4%) |  |
| ≥30 | 2,505 (8.2%) | 264 (10.5%) |  | 992 (9.5%) | 183 (8.1%) |  |
| *Invalid or missing* | 14 183 (46.5%) | 1300 (51.5%) |  | 4124 (39.4%) | 977 (43.2%) |  |
| **Smoking, early pregnancy**^c^ |  |  |  |  |  |  |
| Yes | 6478 (21.2%) | 687 (27.2%) | 0.169 | 4201 (40.1%) | 778 (34.4%) | 0.124 |
| No | 20 394 (66.9%) | 1484 (58.8%) |  | 5963 (57.0%) | 1424 (63.0%) |  |
| Missing | 3628 (11.9%) | 351 (13.9%) |  | 305 (2.9%) | 59 (2.6%) |  |
| **Parity** |  |  |  |  |  |  |
| 0 | 13 883 (45.5%) | 1074 (42.6%) | 0.152 | 4998 (47.7%) | 1072 (47.4%) | 0.009 |
| 1 | 9846 (32.3%) | 723 (28.7%) |  | 3223 (30.8%) | 695 (30.7%) |  |
| ≥ 2 | 6771 (22.2%) | 725 (28.7%) |  | 2248 (21.5%) | 494 (21.8%) |  |
| **Maternal comorbidity** |  |  |  |  |  |  |
| Yes | 1664 (5.5%) | 178 (7.1%) | 0.066 | 420 (4.0%) | 81 (3.6%) | 0.022 |
| **Use of known/suspected teratogens** | |  |  |  |  |  |
| Yes | 2,735 (9.0%) | 259 (10.3%) | 0.044 | 595 (5.7%) | 136 (6.0%) | 0.014 |
| **Other medications during pregnancy** | |  |  |  |  |  |
| Yes | 15 708 (51.5%) | 1310 (51.9%) | 0.009 | 4607 (44.0%) | 896 (39.6%) | 0.089 |
| **Child characteristics** |  |  |  |  |  |  |
| **Child country of birth** |  |  |  |  |  |  |
| Iceland | 153 (0.5%) | 30 (1.2%) | 0.12 | - | - |  |
| Norway | 17 471 (57.3%) | 1549 (61.4%) |  | - | - |  |
| Sweden | 12 876 (42.2%) | 943 (37.4%) |  | - | - |  |
| Denmark | - | - |  | 10 469 (100%) | 2261 (100%) |  |
| **Child sex** |  |  |  |  |  |  |
| Female | 15 053 (49.4%) | 984 (39.0%) | 0.209 | 5051 (48.2%) | 1037 (45.9%) | 0.048 |
| Male | 15 447 (50.6%) | 1538 (61.0%) |  | 5418 (51.8%) | 1224 (54.1%) |  |
| **Calendar year of birth** |  |  |  |  |  |  |
| 2000-2005 | 1,690 (5.5%) | 154 (6.1%) | 0.024 | 5777 (55.2%) | 1328 (58.7%) | 0.072 |
| 2006-2011 | 28,810 (94.5%) | 2,368 (93.9%) |  | 4692 (44.8%) | 933 (41.3%) |  |
| **Paediatric comorbidity index score^d^** | | |  |  |  |  |
| Zero | 17,296 (56.7%) | 1,261 (50.0%) | 0.294 | 7,119 (68.0%) | 1,519 (67.2%) | 0.123 |
| 1-2 | 9,575 (31.4%) | 735 (29.1%) |  | 2,340 (22.4%) | 474 (21.0%) |  |
| 3-5 | 3,102 (10.2%) | 351 (13.9%) |  | 896 (8.6%) | 207 (9.2%) |  |
| >5 | 527 (1.7%) | 175 (6.9%) |  | 114 (1.1%) | 61 (2.7%) |  |
| **Child diagnosis of ADHD ^e^** |  |  |  |  |  |  |
| Yes | 789 (2.6%) | 227 (9.0%) | 0.277 | 250 (2.4%) | 74 (3.3%) | 0.053 |
| **Child fill for psychotropic medication ^f^** |  |  |  |  |  |  |
| Yes | 4,776 (15.7%) | 733 (29.1%) | 0.326 | 470 (4.5%) | 126 (5.6%) | 0.05 |
| **Reason for no test result ^g^** |  |  |  |  |  |  |
| Participated | 29 679 (97.3%) | 0 (0%) | 5.645 | 8897 (85.0%) | - | 4.014 |
| Missing language only | 452 (1.5%) | 0 (0%) |  | 1097 (10.5%) | - |  |
| Missing math only | 369 (1.2%) | 0 (0%) |  | 475 (4.5%) | - |  |
| Absent | - | 381 (15.1%) |  | - | - |  |
| Exempt | - | 812 (32.2%) |  | - | - |  |
| No academic data | - | 985 (39.1%) |  | - | 2037 (90.1%) |  |
| Emigration | - | 190 (7.5%) |  | - | 172 (7.6%) |  |
| Death | - | 154 (6.1%) |  | - | 52 (2.3%) |  |

ADHD, attention-deficit/hyperactive disorder; ATC, anatomical therapeutic chemical classification system; BMI, body mass index; ICD-10, International statistical classification of diseases and related health conditions, revision 10; MBR, Medical birth register; NPR, National patient register; PDR, Prescribed drug register; SMD, standardised mean difference

Finland was not included in this analysis due to data availability.

^a^ Psychiatric disorder recorded as the indication for prescription using reimbursement codes for chronic psychiatric disorders for women in Norway with births between 2005 and 2010.

^b^ In Finland, Iceland, Norway and Sweden, cohabitating refers to any situation where the mother reports living with a partner, in Denmark cohabiting refers to married or registered partnership only in Denmark.

^c^ Not available for Iceland.

^d^ Paediatric comorbidity index score was calculated from 0 (no comorbidity) to 10+ (high comorbidity) based diagnosis records before age 8 in the NPR & MBR using ICD-10 codes for 24 predefined and empirically identified conditions.

^e^ Record of at least one ADHD (ICD-10 code: F90) diagnosis before age 8 years in the NPR.

^f^ Record of at least one psychotropic medication (ATC code: N0) before age 8 years in the PDR.

^g^ Not all reasons were available for every country.

eTable S10. Inverse probability of selection (censoring) analysis: risk ratios (with 95% confidence intervals) for poor academic performance after prenatal antipsychotic exposure by timing of exposure and monotherapy.

|  |  | **Risk Ratio (95% CI)** | | |
| --- | --- | --- | --- | --- |
|  | **No of events/No of pregnancies** | **Minimally adjusted ^a^** | **Fully adjusted ^b^** | **Fully adjusted ^b^** |
| **Mathematics** |  |  |  |  |
| Unexposed | 13 190/38 088 | Reference | Reference | 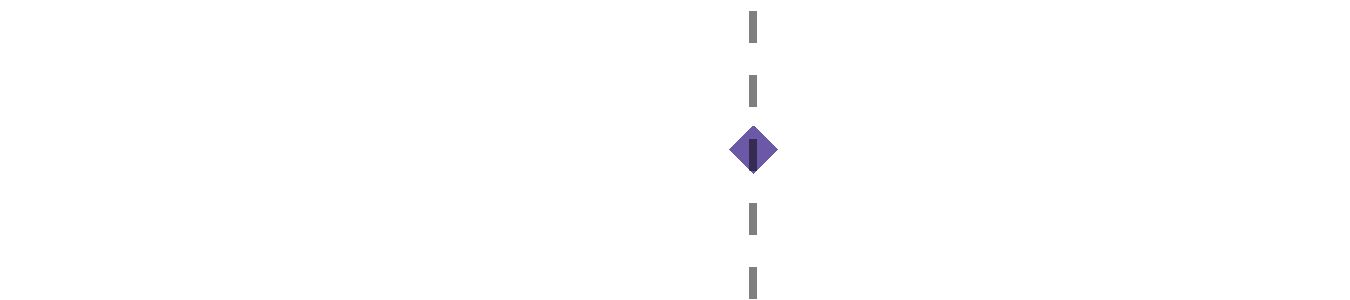 |
| Exposed any time | 751/1906 | 1.17 (1.10 – 1.25) | 1.04 (0.95 – 1.14) | 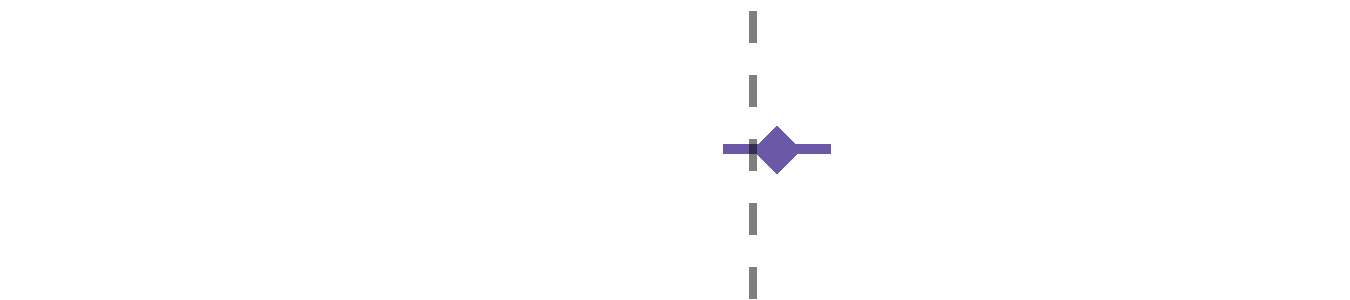 |
| Exposed in late pregnancy only | 132/375 | 1.07 (0.92 – 1.25) | 0.95 (0.77 – 1.17) | 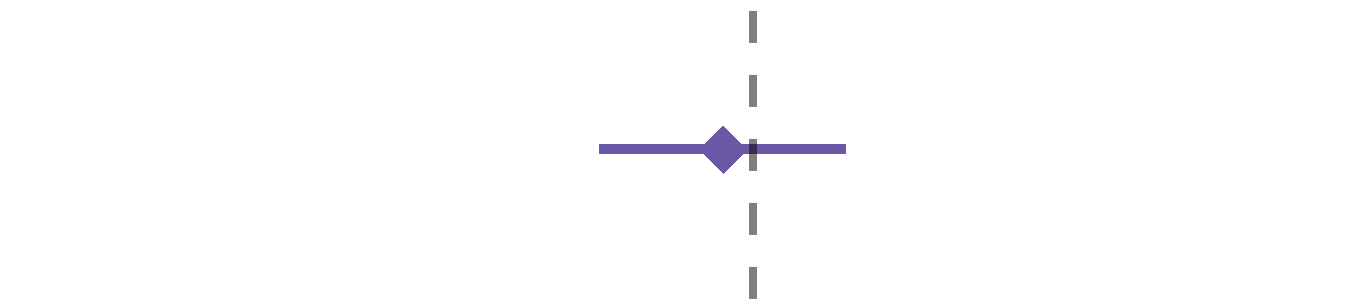 |
| Exposed throughout pregnancy | 286/668 | 1.29 (1.17 – 1.43) | 1.12 (0.97 – 1.31) | 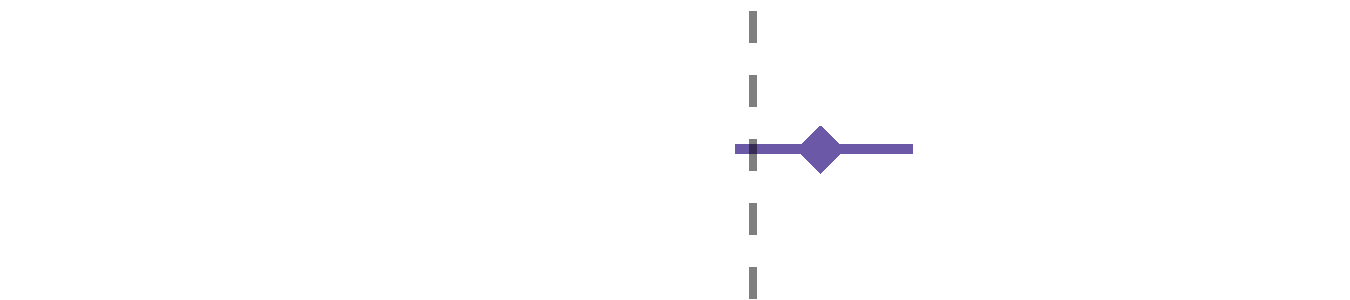 |
| Olanzapine | 115/281 | 1.20 (1.02 – 1.41) | 1.05 (0.82 – 1.35) | 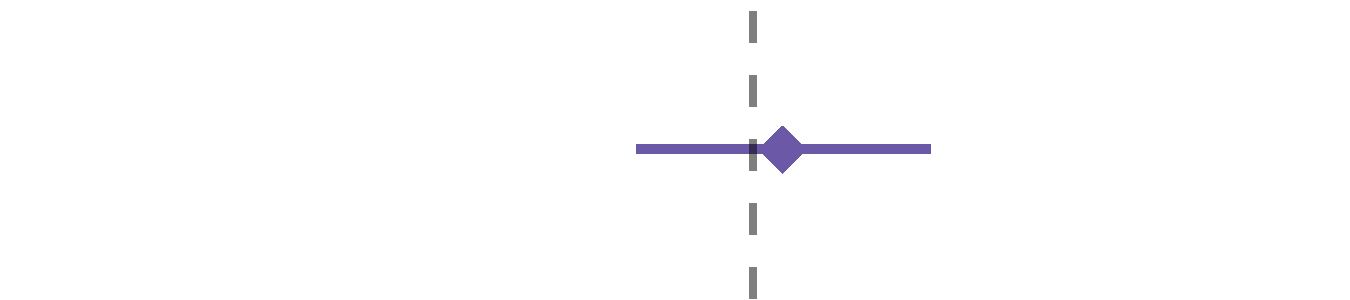 |
| Quetiapine | 83/199 | 1.24 (1.03 – 1.49) | 1.09 (0.85 – 1.40) | 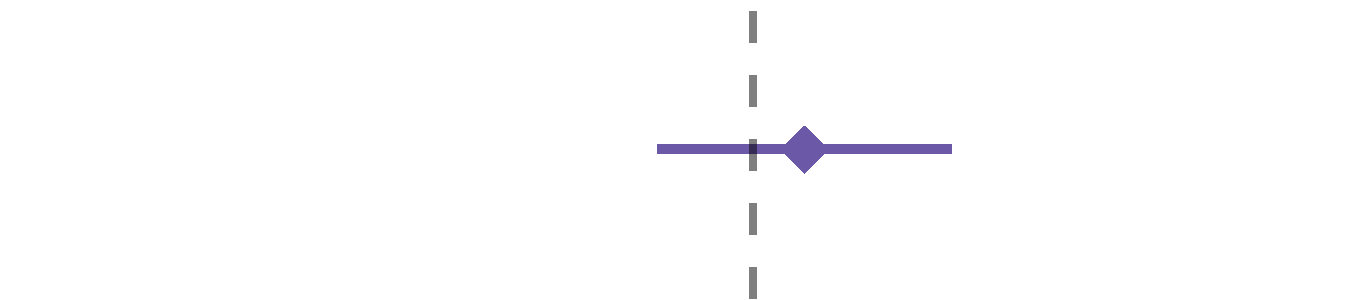 |
| Chlorprothixene | 70/170 | 1.25 (1.03 – 1.52) | 1.11 (0.89 – 1.39) | 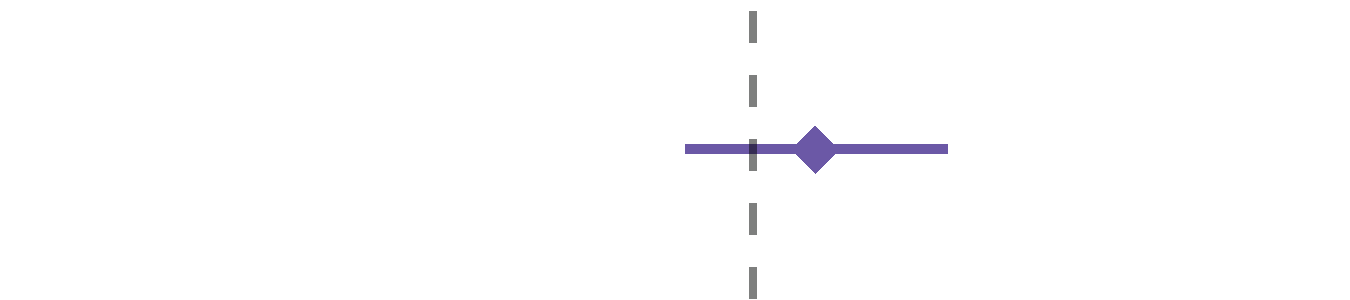 |
| Prochlorperazine | 53/167 | 0.97 (0.75 – 1.25) | 0.92 (0.65 – 1.31) | 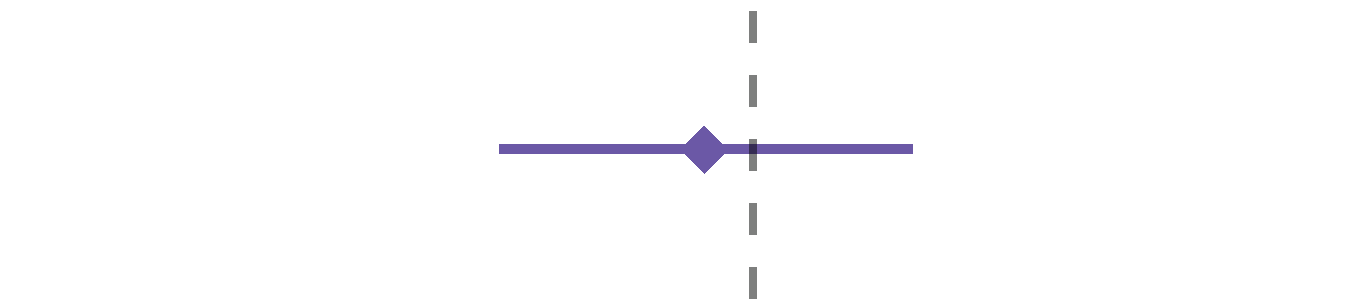 |
| Perphenazine | 68/164 | 1.23 (1.00 – 1.52) | 1.09 (0.80 – 1.48) | 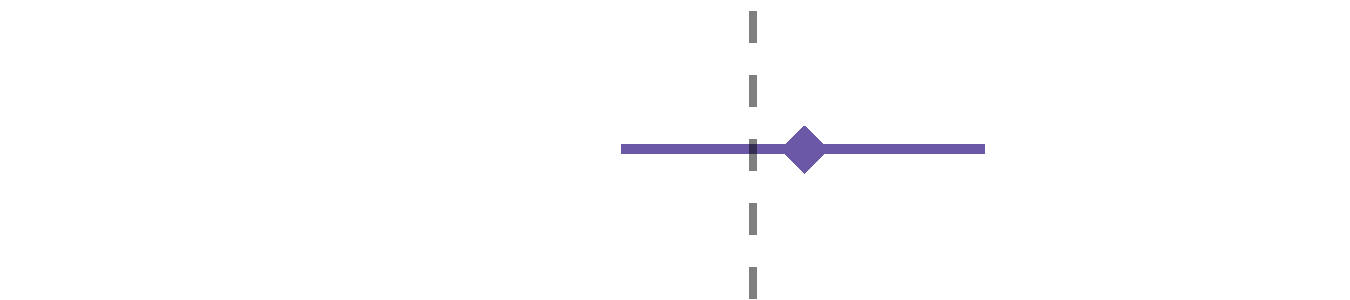 |
| Levomepromazine | 71/160 | 1.38 (1.12 – 1.7) | 1.20 (0.91 – 1.59) | 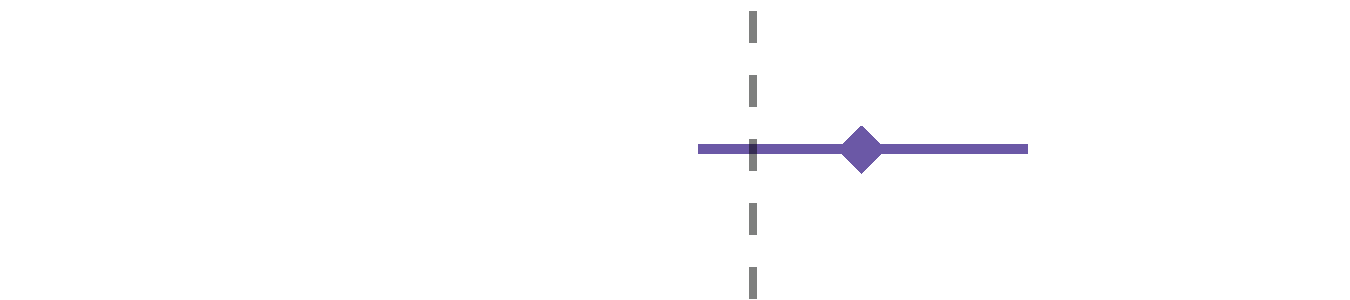 |
| Risperidone | 27/68 | 1.14 (0.81 – 1.59) | 1.05 (0.69 – 1.62) | 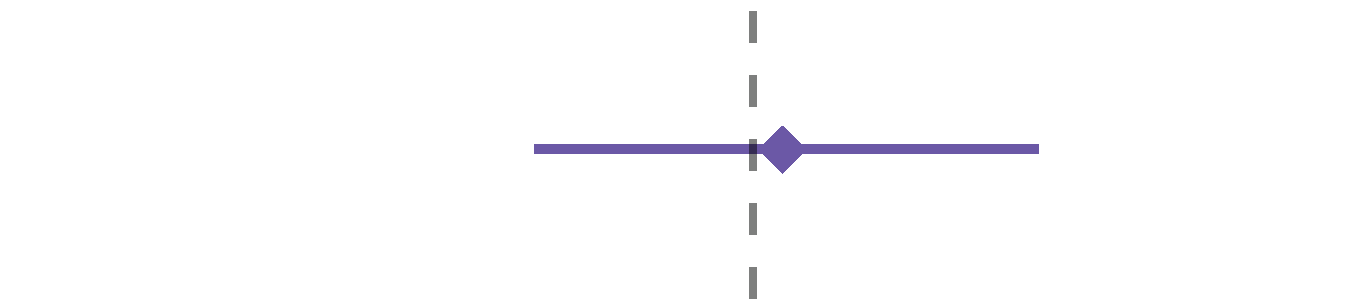 |
| Dixyrazine | 31/67 | 1.16 (0.83 – 1.61) | 1.14 (0.70 – 1.85) | 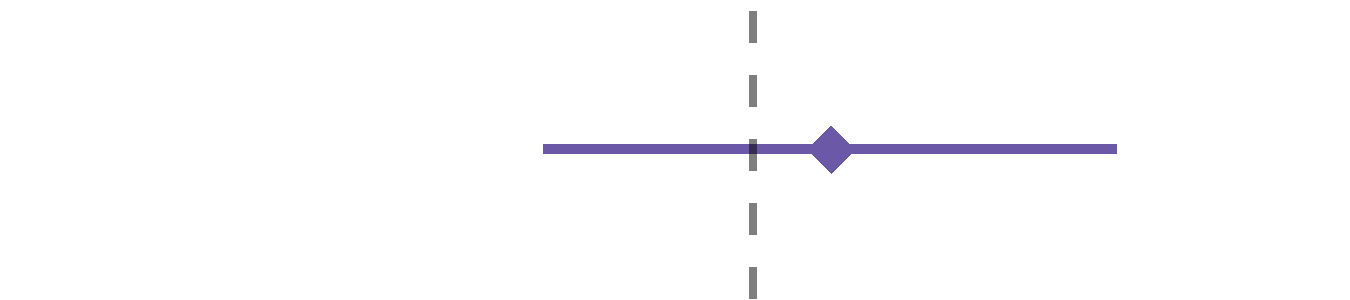 |
| Chlorpromazine | 19/65 | 0.94 (0.62 – 1.43) | 0.91 (0.51 – 1.63) | 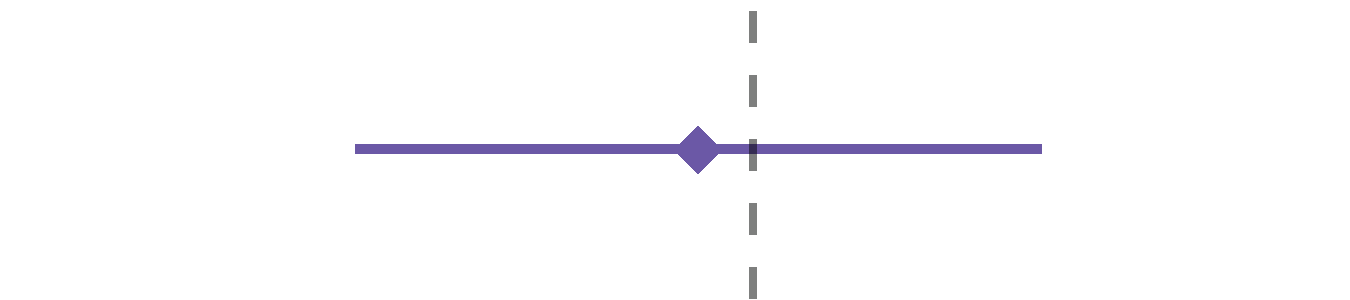 |
| Flupentixol | 18/49 | 1.08 (0.70 – 1.67) | 0.99 (0.54 – 1.83) | 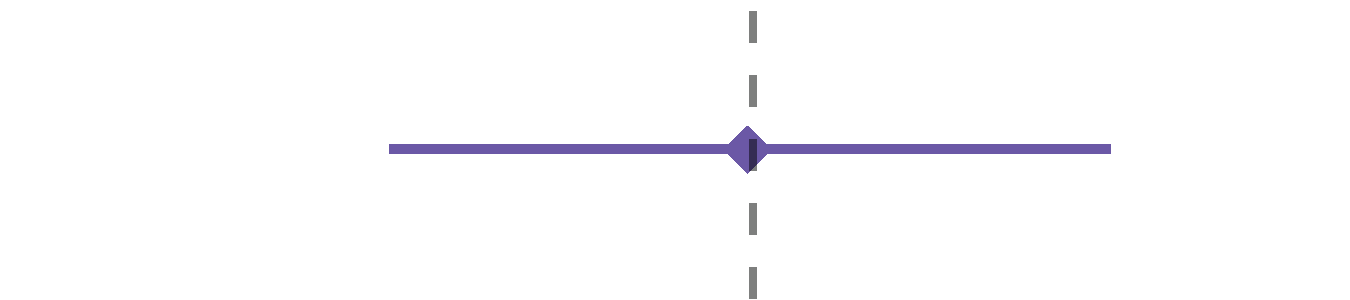 |
| Aripiprazole | 15/31 | 1.33 (0.82 – 2.15) | 1.19 (0.57 – 2.47) | 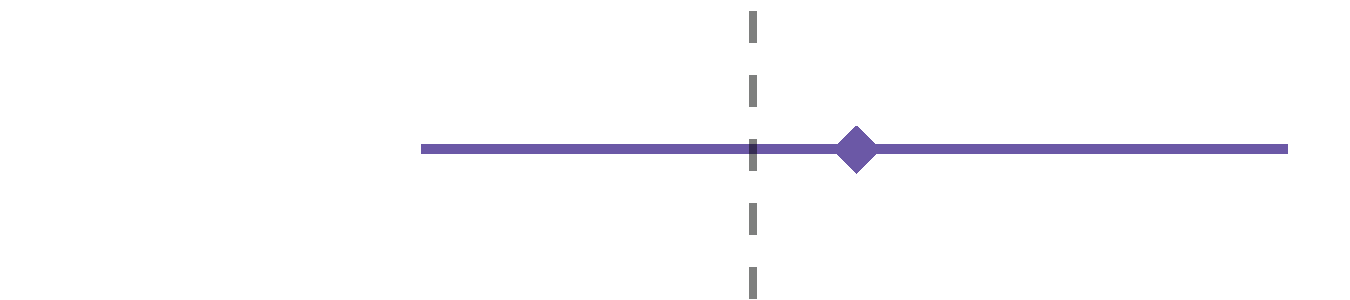 |
| **Language Arts** |  |  |  |  |
| Unexposed | 11 105/37 541 | Reference | Reference | 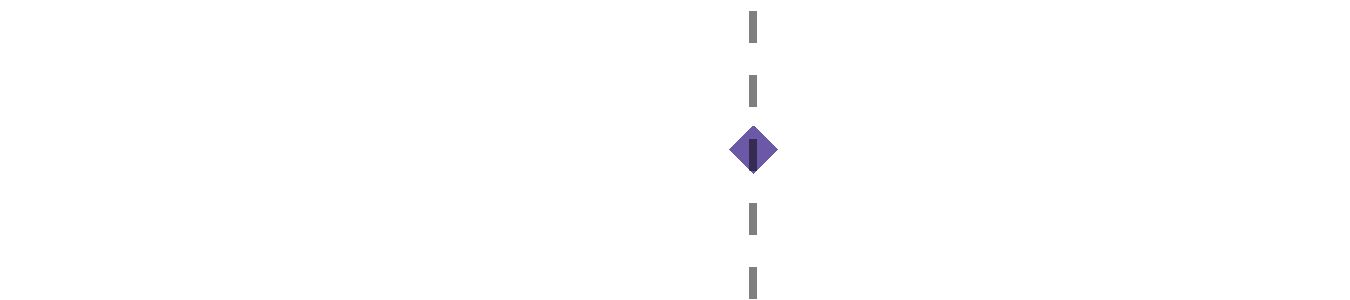 |
| Exposed any time | 625/1879 | 1.13 (1.05 – 1.21) | 1.00 (0.91 – 1.11) | 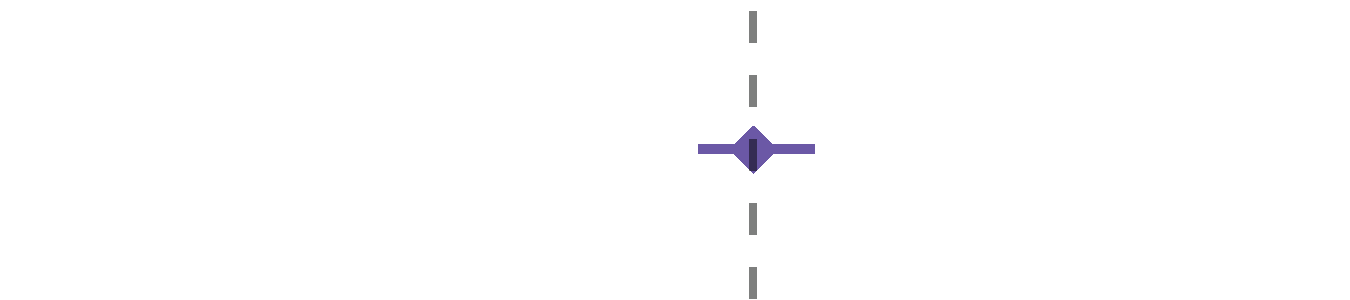 |
| Exposed in late pregnancy only | 113/362 | 1.04 (0.88 – 1.22) | 0.95 (0.75 – 1.19) | 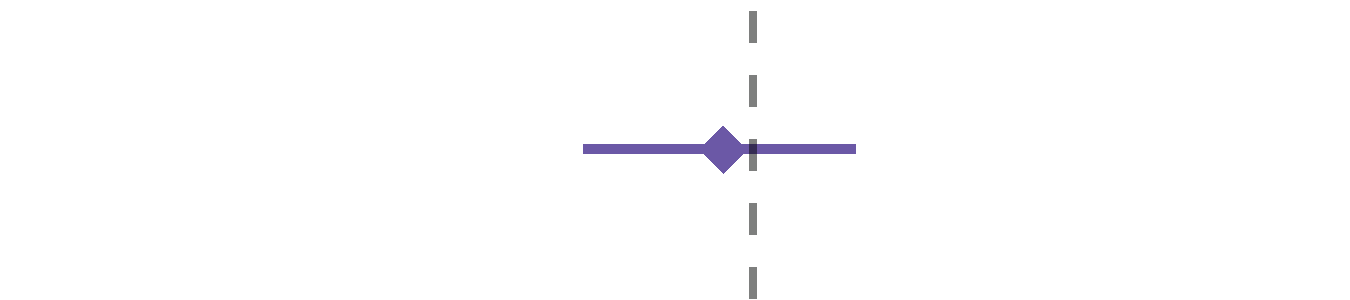 |
| Exposed throughout pregnancy | 227/662 | 1.19 (1.06 – 1.34) | 1.08 (0.92 – 1.27) | 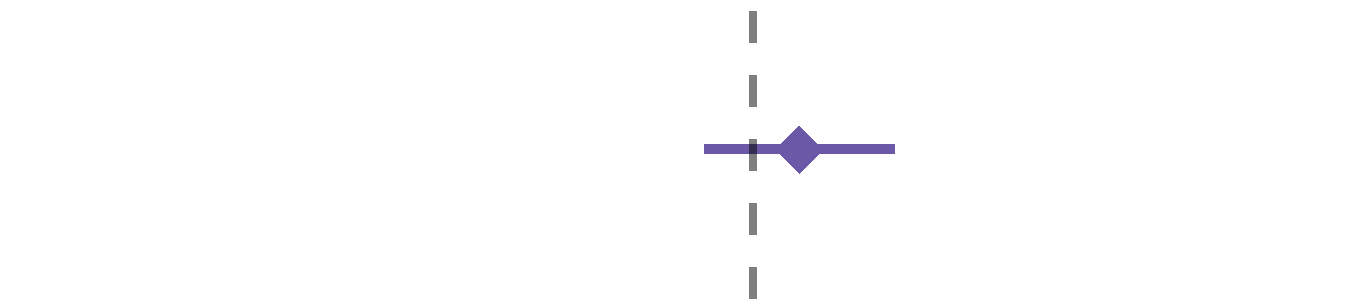 |
| Olanzapine | 86/275 | 1.11 (0.92 – 1.35) | 1.00 (0.76 – 1.32) | 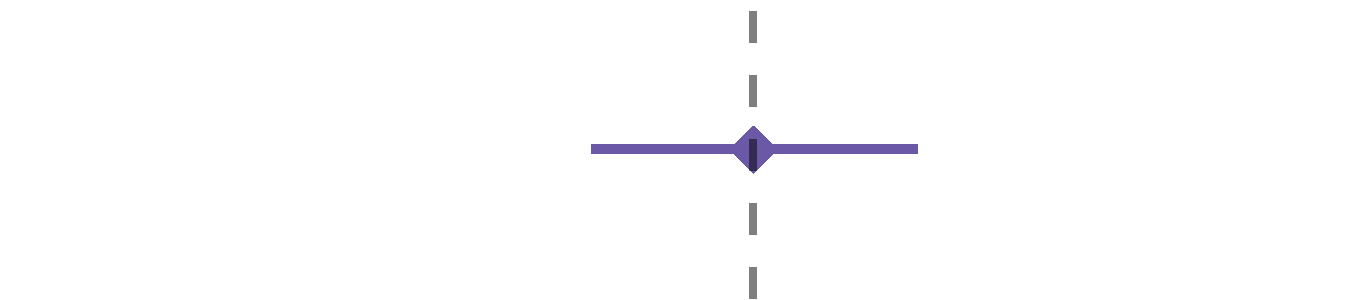 |
| Quetiapine | 65/198 | 1.12 (0.91 – 1.37) | 1.12 (0.86 – 1.46) | 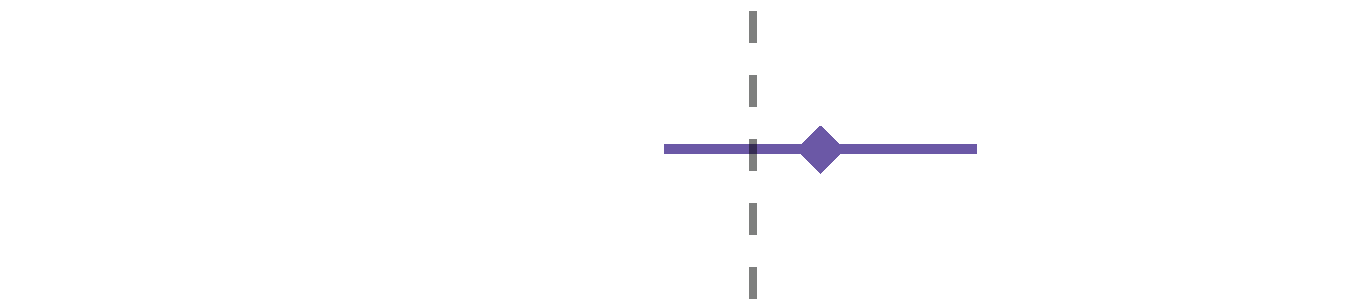 |
| Chlorprothixene | 66/168 | 1.32 (1.08 – 1.61) | 1.13 (0.88 – 1.44) | 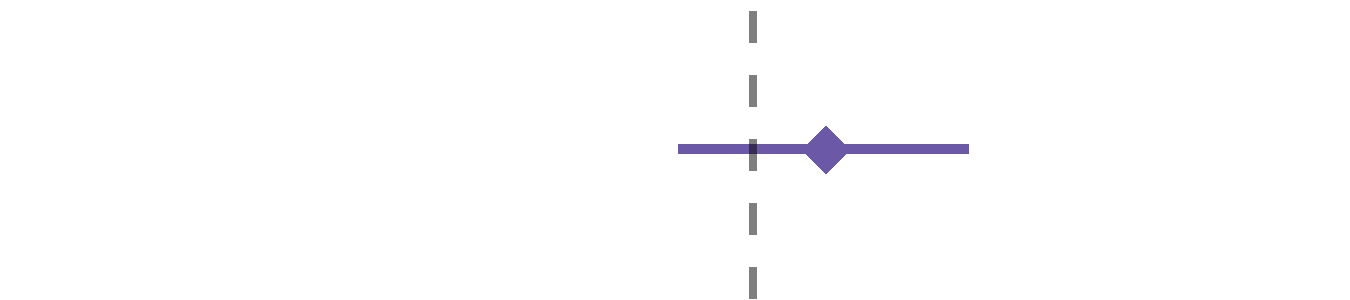 |
| Prochlorperazine | 50/167 | 1.02 (0.78 – 1.33) | 0.98 (0.68 – 1.42) | 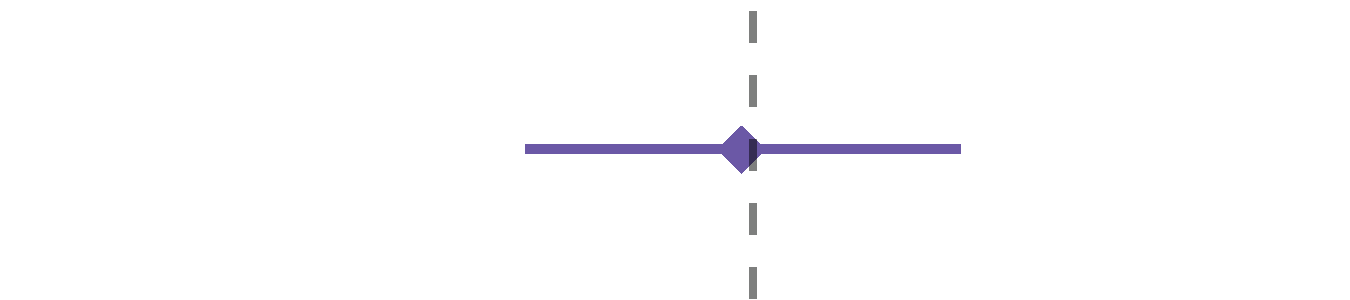 |
| Perphenazine | 55/159 | 1.14 (0.91 – 1.43) | 1.07 (0.77 – 1.47) | 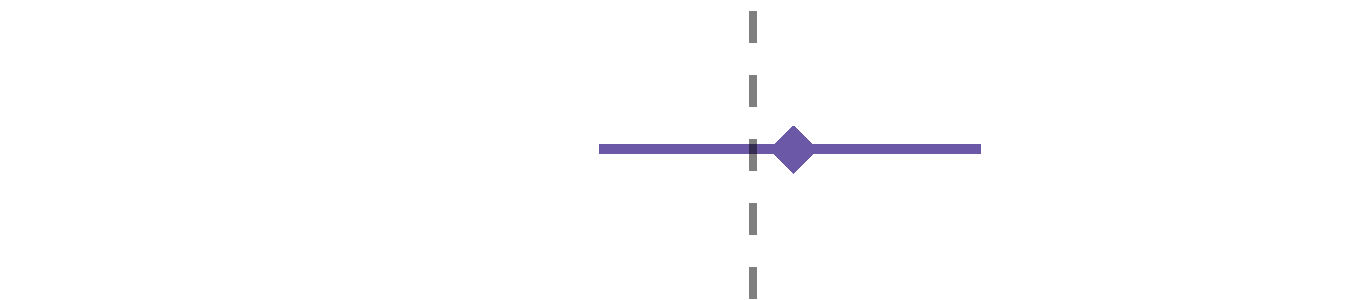 |
| Levomepromazine | 54/157 | 1.18 (0.92 – 1.51) | 1.03 (0.74 – 1.42) | 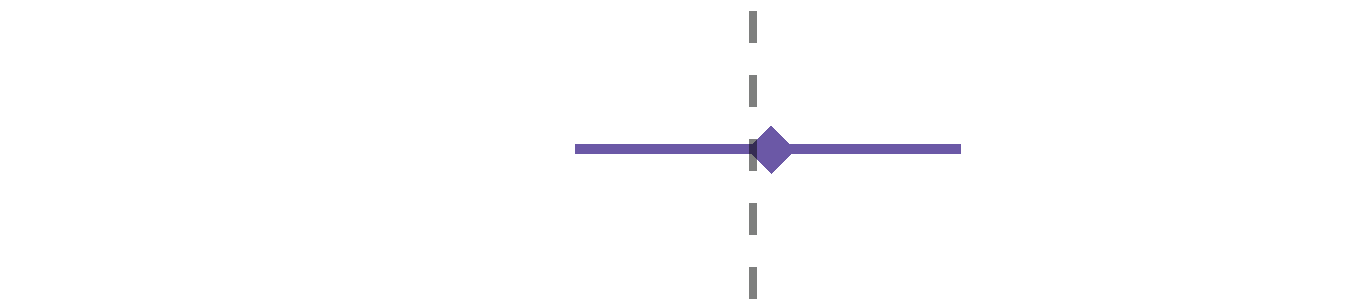 |
| Risperidone | 19/69 | 0.97 (0.64 – 1.47) | 0.77 (0.45 – 1.33) | 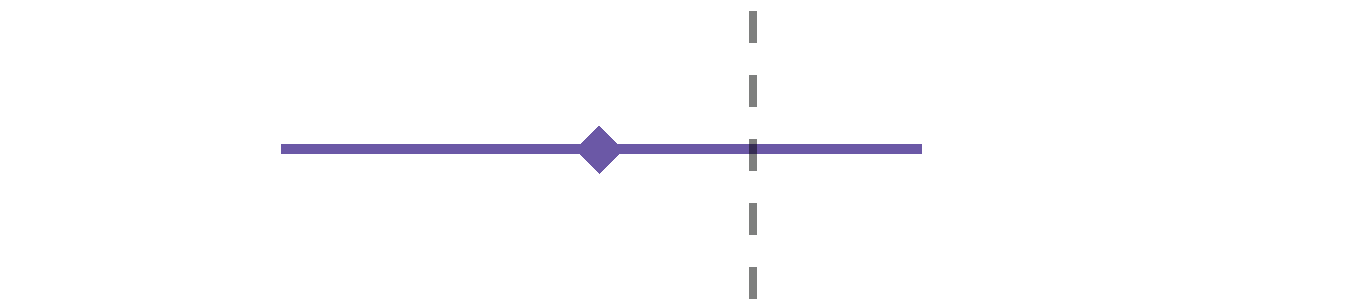 |
| Dixyrazine | 21/66 | 1.07 (0.72 – 1.59) | 1.01 (0.57 – 1.77) | 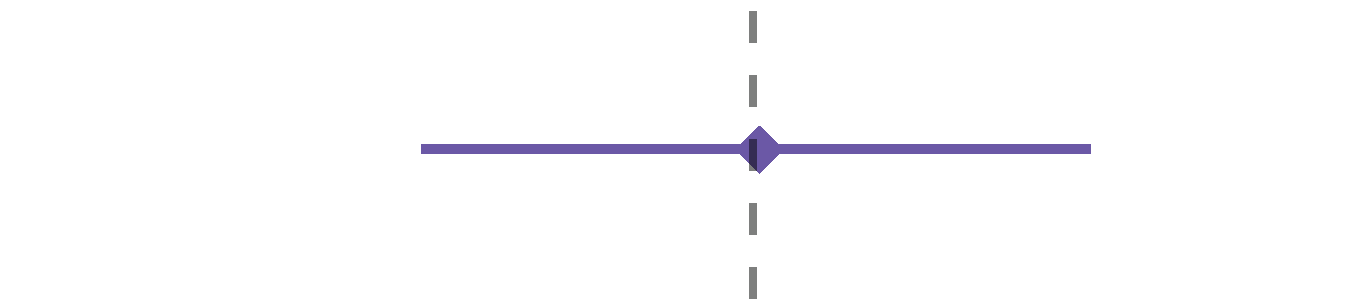 |
| Chlorpromazine | 24/66 | 1.27 (0.88 – 1.84) | 1.22 (0.70 – 2.13) | 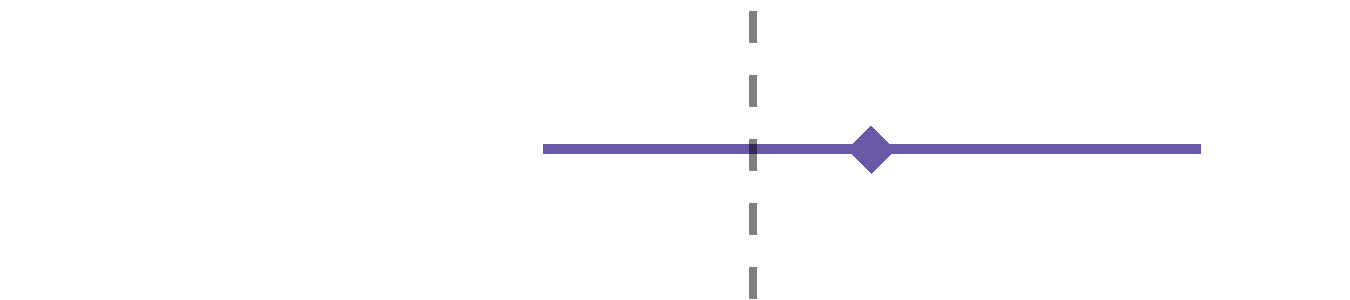 |
| Flupentixol | 13/49 | 1.00 (0.60 – 1.65) | 0.91 (0.45 – 1.83) | 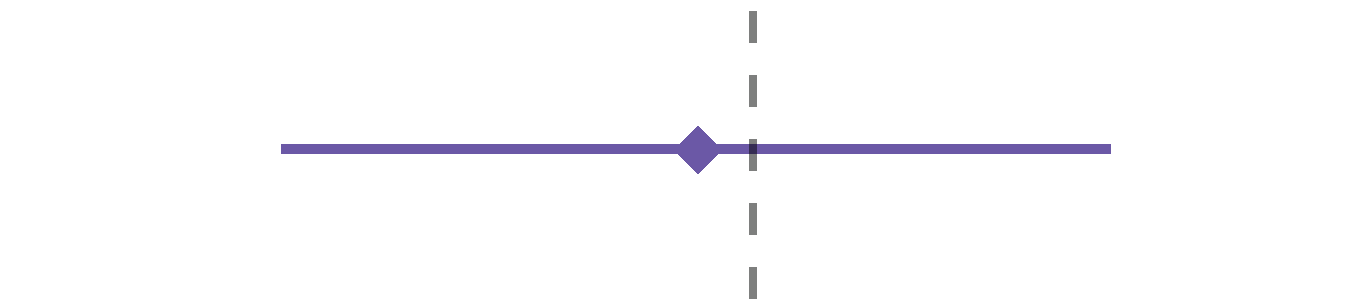 |
| Aripiprazole | 8/32 | 0.88 (0.46 – 1.68) | 0.74 (0.31 – 1.75) | 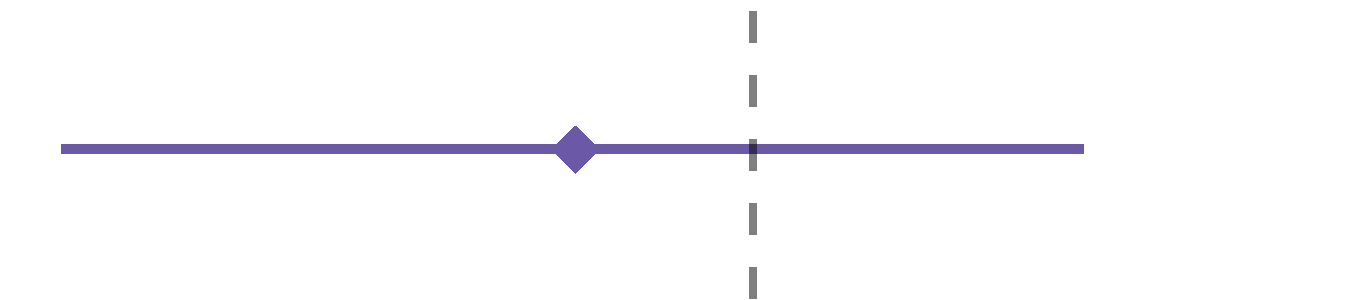 |
|  |  |  |  | 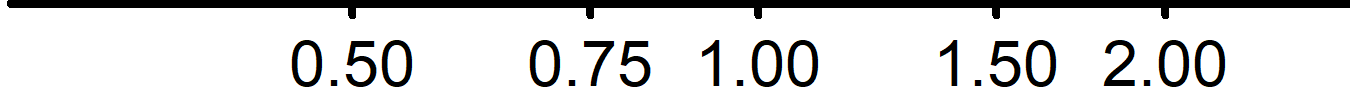 |

ADHD, attention-deficit/hyperactive disorder; ATC, anatomical therapeutic chemical classification system; BMI, body mass index; CI, confidence interval; ICD-10, International statistical classification of diseases and related health conditions, revision 10; MBR, Medical birth register; NPR, National patient register; PDR, Prescribed drug register

Finland was not included in this analysis due to data availability.

Inverse probability of selection (censoring) weights was created using sex of child, paediatric comorbidity index score, ADHD diagnosis and psychotropic use before age 8 years and maternal education and maternal birth country. Paediatric comorbidity index score was calculated from 0 (no comorbidity) to 10+ (high comorbidity) based diagnosis records before age 8 in the NPR & MBR using ICD-10 codes for 24 predefined and empirically-identified conditions ^8^, ADHD diagnosis was defined as a record of at least one ADHD (ICD-10 code: F90) diagnosis before age 8 years in the NPR and psychotropic medication use was defined as a record of at least one psychotropic medication (ATC code: N0) before age 8 years in the PDR.

^a^ Adjusted for birth year and sex of child, child´s country of birth, maternal age, parity using outcome regression and also for likelihood of no school test results using inverse probability selection (censoring) weights.

^b^ Adjusted for birth year, sex of child, child´s country of birth, maternal country of birth, age, parity, education, cohabitation status, BMI & smoking in early pregnancy, use of other medications during pregnancy, or known/suspected teratogens and comorbidity prior to pregnancy using propensity score overlap weights and also for likelihood of no school test results using inverse probability selection (censoring) weights. These weights were multiplied together.

eTable S11. Post-hoc analysis: hazard ratios (with 95% confidence intervals) for child neurodevelopmental disorders when comparing prenatal chlorpromazine monotherapy exposure to women who discontinued antipsychotic treatment before pregnancy.

|  | Exposed in pre-pregnancy only | Exposed any time during | Hazard Ratio (95% CI) | | |
| --- | --- | --- | --- | --- | --- |
|  | No of events/No of pregnancies | No of events/No of pregnancies | Minimally adjusted ^a^ | Fully adjusted ^b^ | Fully adjusted ^b^ |
| Developmental intellectual disorder | 16/4908 | <5/153 | 2.54 (0.54 – 11.92) | 3.33 (0.91 – 12.14) | 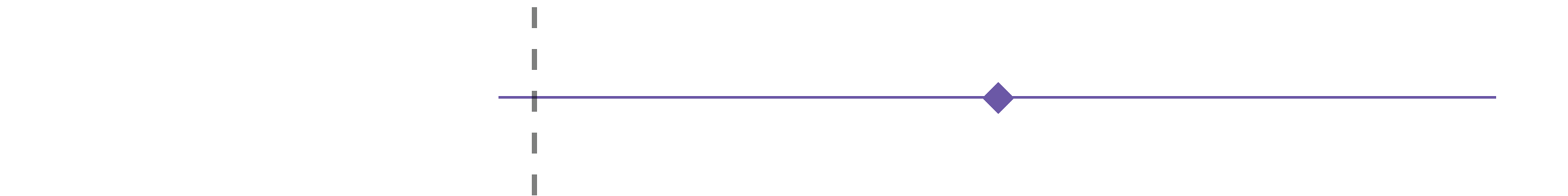 |
| Learning disorder | 29/4908 | <5/153 | 1.37 (0.43 – 4.44) | 1.18 (0.34 – 4.12) | 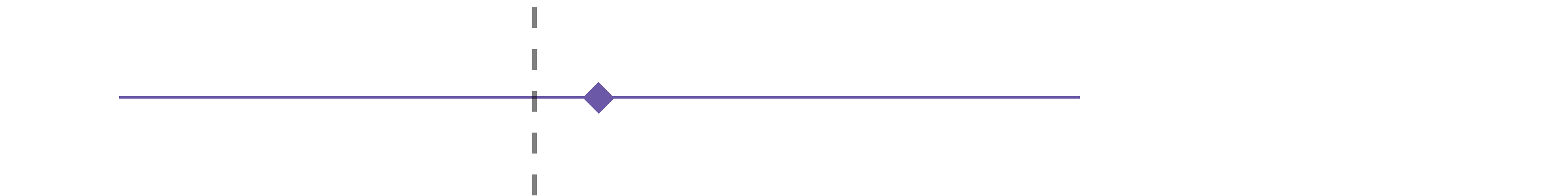 |
| Speech or language disorder | 33/4908 | <5/153 | 2.59 (0.77 – 8.71) | 2.46 (0.80 – 7.54) | 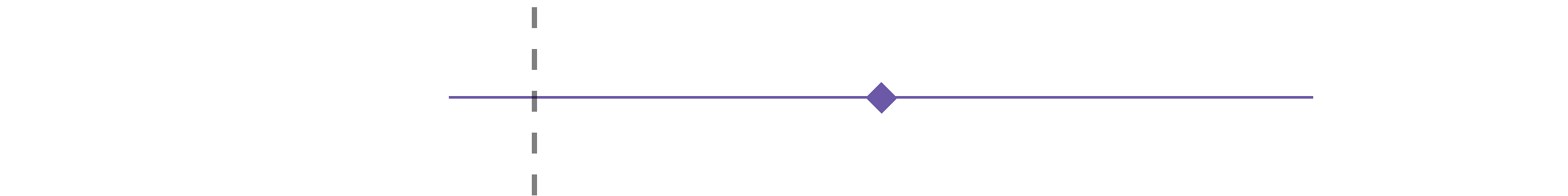 |
| Composite neurodevelopmental outcome | 74/4908 | 10/153 | 2.20 (1.08 – 4.50) | 2.17 (1.09 – 4.35) | 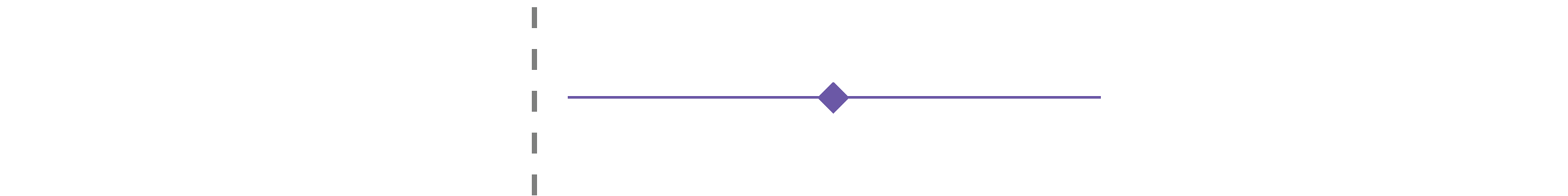 |
|  |  |  |  |  | 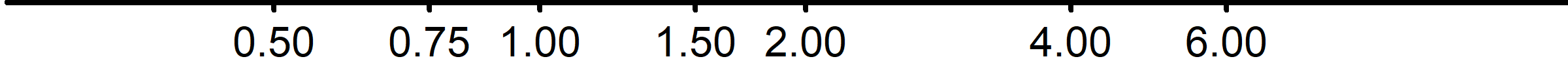 |

BMI, body mass index; CI, confidence interval

Finland was not included in this analysis due to data availability and Denmark was not included due to low numbers.

^a^ Adjusted for birth year and sex of child, child´s country of birth, maternal age, parity using outcome regression.

^b^ Adjusted for birth year, sex of child, child´s country of birth of child, maternal country of birth, age, parity, education, cohabitation status, BMI & smoking in early pregnancy, use of other medications during pregnancy, or known/suspected teratogens and comorbidity prior to pregnancy using propensity score overlap weights.

eTable S12. REporting of studies Conducted using Observational Routinely collected health Data (RECORD) reporting guidelines for observational studies.^9^

| Item No | STROBE items | RECORD items | RECORD-PE items | Page No |
| --- | --- | --- | --- | --- |
| **Title and abstract** | | | | |
| 1 | (a) Indicate the study’s design with a commonly used term in the title or the abstract.  (b) Provide in the abstract an informative and balanced summary of what was done and what was found. | 1.1: The type of data used should be specified in the title or abstract. When possible, the name of the databases used should be included.  1.2: If applicable, the geographical region and timeframe within which the study took place should be reported in the title or abstract.  1.3: If linkage between databases was conducted for the study, this should be clearly stated in the title or abstract. | — | 1,5,6 |
| **Introduction** | | | | |
| Background rationale | | | | |
| 2 | Explain the scientific background and rationale for the investigation being reported. | — | — | 7 |
| Objectives | | | | |
| 3 | State specific objectives, including any prespecified hypotheses. | — | — | 7 |
| **Methods** | | | | |
| Study design | | | | |
| 4 | Present key elements of study design early in the paper. | — | 4.a: Include details of the specific study design (and its features) and report the use of multiple designs if used.  4.b: The use of a diagram(s) is recommended to illustrate key aspects of the study design(s), including exposure, washout, lag and observation periods, and covariate definitions as relevant. | 8 |
| Setting | | | | |
| 5 | Describe the setting, locations, and relevant dates, including periods of recruitment, exposure, follow-up, and data collection. | — | — | 8, 9, eAppendix |
| Participants | | | | |
| 6 | (a) Cohort study—give the eligibility criteria, and the sources and methods of selection of participants. Describe methods of follow-up. Case-control study—give the eligibility criteria, and the sources and methods of case ascertainment and control selection. Give the rationale for the choice of cases and controls. Cross sectional study—give the eligibility criteria, and the sources and methods of selection of participants.  (b) Cohort study—for matched studies, give matching criteria and number of exposed and unexposed. Case-control study—for matched studies, give matching criteria and the number of controls per case. | 6.1: The methods of study population selection (such as codes or algorithms used to identify participants) should be listed in detail. If this is not possible, an explanation should be provided.  6.2: Any validation studies of the codes or algorithms used to select the population should be referenced. If validation was conducted for this study and not published elsewhere, detailed methods and results should be provided.  6.3: If the study involved linkage of databases, consider use of a flow diagram or other graphical display to demonstrate the data linkage process, including the number of individuals with linked data at each stage. | 6.1.a: Describe the study entry criteria and the order in which these criteria were applied to identify the study population. Specify whether only users with a specific indication were included and whether patients were allowed to enter the study population once or if multiple entries were permitted. See explanatory document for guidance related to matched designs. | 8,9,10 eTable 3 |
| Variables | | | | |
| 7 | Clearly define all outcomes, exposures, predictors, potential confounders, and effect modifiers. Give diagnostic criteria, if applicable. | 7.1: A complete list of codes and algorithms used to classify exposures, outcomes, confounders, and effect modifiers should be provided. If these cannot be reported, an explanation should be provided. | 7.1.a: Describe how the drug exposure definition was developed.  7.1.b: Specify the data sources from which drug exposure information for individuals was obtained.  7.1.c: Describe the time window(s) during which an individual is considered exposed to the drug(s). The rationale for selecting a particular time window should be provided. The extent of potential left truncation or left censoring should be specified.  7.1.d: Justify how events are attributed to current, prior, ever, or cumulative drug exposure.  7.1.e: When examining drug dose and risk attribution, describe how current, historical or time on therapy are considered.  7.1.f: Use of any comparator groups should be outlined and justified.  7.1.g: Outline the approach used to handle individuals with more than one relevant drug exposure during the study period. | 9,10,12  eTable 3 |
| Data sources/measurement | | | | |
| 8 | For each variable of interest, give sources of data and details of methods of assessment (measurement). Describe comparability of assessment methods if there is more than one group. | — | 8.a: Describe the healthcare system and mechanisms for generating the drug exposure records. Specify the care setting in which the drug(s) of interest was prescribed. | 8  eAppendix |
| Bias | | | | |
| 9 | Describe any efforts to address potential sources of bias. | — | — | 8 - 12 |
| Study size | | | | |
| 10 | Explain how the study size was arrived at. | — | — | 8 eFigure 1 |
| Quantitative variables | | | | |
| 11 | Explain how quantitative variables were handled in the analyses. If applicable, describe which groupings were chosen, and why. | — | — | eTable 3 |
| Statistical methods | | | | |
| 12 | (a) Describe all statistical methods, including those used to control for confounding.  (b) Describe any methods used to examine subgroups and interactions.  (c) Explain how missing data were addressed.  (d) Cohort study—if applicable, explain how loss to follow-up was addressed. Case-control study—if applicable, explain how matching of cases and controls was addressed. Cross sectional study—if applicable, describe analytical methods taking account of sampling strategy.  (e) Describe any sensitivity analyses. | — | 12.1.a: Describe the methods used to evaluate whether the assumptions have been met.  12.1.b: Describe and justify the use of multiple designs, design features, or analytical approaches. | 11, 12  eTable 4 |
| Data access and cleaning methods | | | | |
| 12 | — | 12.1: Authors should describe the extent to which the investigators had access to the database population used to create the study population.  12.2: Authors should provide information on the data cleaning methods used in the study. | — | 8, 20 |
| Linkage | | | | |
| 12 | — | 12.3: State whether the study included person level, institutional level, or other data linkage across two or more databases. The methods of linkage and methods of linkage quality evaluation should be provided. | — | NA |
| **Results** | | | | |
| Participants | | | | |
| 13 | (a) Report the numbers of individuals at each stage of the study (e.g., numbers potentially eligible, examined for eligibility, confirmed eligible, included in the study, completing follow-up, and analysed).  (b) Give reasons for non-participation at each stage.  (c) Consider use of a flow diagram. | 13.1: Describe in detail the selection of the individuals included in the study (that is, study population selection) including filtering based on data quality, data availability, and linkage. The selection of included individuals can be described in the text or by means of the study flow diagram. | — | 13 eFigure 1 |
| Descriptive data | | | | |
| 14 | (a) Give characteristics of study participants (e.g., demographic, clinical, social) and information on exposures and potential confounders.  (b) Indicate the number of participants with missing data for each variable of interest.  (c) Cohort study—summarise follow-up time (e.g., average and total amount). | — | — | 13, 14  Table 1,  eTable 5 |
| Outcome data | | | | |
| 15 | Cohort study—report numbers of outcome events or summary measures over time. Case-control study—report numbers in each exposure category, or summary measures of exposure. Cross sectional study—report numbers of outcome events or summary measures. | — | — | Figure 1,  Figure 2,  Figure 4,  eTable 5 |
| Main results | | | | |
| 16 | (a) Give unadjusted estimates and, if applicable, confounder adjusted estimates and their precision (e.g., 95% confidence intervals). Make clear which confounders were adjusted for and why they were included.  (b) Report category boundaries when continuous variables are categorised.  (c) If relevant, consider translating estimates of relative risk into absolute risk for a meaningful time period. | — | — | (a) 14  Figure 1, Figure 2  (b) NA (c) eTable 5 |
| Other analyses | | | | |
| 17 | Report other analyses done—e.g., analyses of subgroups and interactions, and sensitivity analyses. | — | — | 15,16  Figure 3  eTable 6-11 |
| **Discussion** | | | | |
| Key results | | | | |
| 18 | Summarise key results with reference to study objectives. | — | — | 16 |
| Limitations | | | | |
| 19 | Discuss limitations of the study, taking into account sources of potential bias or imprecision. Discuss both direction and magnitude of any potential bias. | 19.1: Discuss the implications of using data that were not created or collected to answer the specific research question(s). Include discussion of misclassification bias, unmeasured confounding, missing data, and changing eligibility over time, as they pertain to the study being reported. | 19.1.a: Describe the degree to which the chosen database(s) adequately captures the drug exposure(s) of interest. | 18 |
| Interpretation | | | | |
| 20 | Give a cautious overall interpretation of results considering objectives, limitations, multiplicity of analyses, results from similar studies, and other relevant evidence. | — | 20.a: Discuss the potential for confounding by indication, contraindication or disease severity or selection bias (healthy adherer/sick stopper) as alternative explanations for the study findings when relevant. | 16, 17, 18, 19 |
| Generalisability | | | | |
| 21 | Discuss the generalisability (external validity) of the study results. | — | — | 17 |
| **Other information** | | | | |
| Funding | | | | |
| 22 | Give the source of funding and the role of the funders for the present study and, if applicable, for the original study on which the present article is based. | — | — | 21 |
| Accessibility of protocol, raw data, and programming code | | | | |
| 22 | — | 22.1: Authors should provide information on how to access any supplemental information such as the study protocol, raw data, or programming code. | — | 20 |

RECORD, reporting of studies conducted using observational routinely collected data; RECORD-PE, RECORD for pharmacoepidemiological research; STROBE, strengthening the reporting of observational studies in epidemiology.

eReferences

1. Cohen JM, Cesta CE, Kjerpeseth L et al (2021) A common data model for harmonization in the Nordic Pregnancy Drug Safety Studies (NorPreSS). *Norsk Epidemiologi* 29: 1–2. doi: 10.5324/nje.v29i1-2.4053

2. Bliddal M, Broe A, Pottegård A, Olsen J and Langhoff-Roos J (2018) The Danish Medical Birth Register. *Eur J Epidemiol* 33(1): 27-36. doi: 10.1007/s10654-018-0356-1

3. Pottegård A, Schmidt SaJ, Wallach-Kildemoes H, Sørensen HT, Hallas J and Schmidt M (2016) Data Resource Profile: The Danish National Prescription Registry. *Int J Epidemiol* 46(3): 798-798f. doi: 10.1093/ije/dyw213

4. Schmidt M, Schmidt SaJ, Sandegaard JL, Ehrenstein V, Pedersen L and Sørensen HT (2015) The Danish National Patient Registry: a review of content, data quality, and research potential. *Clin Epidemiol* 7: 449-490. doi: 10.2147/CLEP.S91125

5. White IR, Royston P and Wood AM (2011) Multiple imputation using chained equations: issues and guidance for practice. *Stat Med* 30(4): 377–399. doi: 10.1002/sim.4067

6. Toutenburg H (1990) *Rubin, D.B.: Multiple imputation for nonresponse in surveys.* New York: Wiley.

7. Buuren SV and Groothuis-Oudshoorn K (2011) mice: Multivariate Imputation by Chained Equations in R. *Journal of Statistical Software* 45(3). doi: 10.18637/jss.v045.i03

8. Sun JW, Bourgeois FT, Haneuse S et al (2021) Development and Validation of a Pediatric Comorbidity Index. *Am J Epidemiol* 190(5): 918–927. doi: 10.1093/aje/kwaa244

9. Langan SM, Schmidt SaJ, Wing K et al (2018) The reporting of studies conducted using observational routinely collected health data statement for pharmacoepidemiology (RECORD-PE). *BMJ* 363: k3532. doi: 10.1136/bmj.k3532
